# WSD-YOLO: A lightweight YOLO-based model with enhanced feature representation for maize pest detection

**DOI:** 10.1371/journal.pone.0357034

**Published:** 2026-09-15

**Authors:** Shenming Qu, Zhiheng Liu, Huazhen Zhao, Yichao Wu, Yang Yang

**Affiliations:** 1 School of Software, Henan University, Kaifeng, China; 2 School of Computer and Information Engineering, Henan University, Kaifeng, China; National Taichung University of Science and Technology, TAIWAN

## Abstract

As a major staple crop with global significance, maize is highly vulnerable to pest infestations throughout its growth cycle, which can substantially limit plant development and reduce yield. In practice, accurate detection remains challenging due to pronounced morphological variations across pest life stages, as well as interference from complex field backgrounds, often leading to missed detections. To address these challenges, we propose WSD-YOLO (YOLOv11n with WindmillConv, Single-Scale High-Level Transformer, and Dual-Attention Weighted Aggregation), a high-accuracy maize pest detection model built upon an improved YOLOv11n framework. The WindmillConv (WMConv) module enhances the model’s sensitivity to multi-directional pest textures, thereby improving low-level feature representation while maintaining a lightweight convolutional design. At a deeper level, the Single-Scale High-Level Transformer (SHLT) introduces global self-attention, enabling effective suppression of background noise with minimal computational overhead. In addition, the Dual-Attention Weighted Aggregation (DAWA) module adaptively fuses same-scale features, improving the detection of pests with diverse morphological characteristics. Experimental results from three repeated training runs (random seeds: 42, 2026, 3407) on the IPMaize dataset demonstrate that the proposed method achieves an averaged mAP@0.5 of 78.77% (79.7% with seed = 42) and averaged mAP@0.5:0.95 of 52.63%. Cross-dataset evaluations on the Tomato Pest&Diseases and IP102 datasets further confirm the model’s strong generalization capability.

## 1. Introduction

Maize is a globally significant food crop and represents the most widely cultivated grain in China, with an annual planting area exceeding $4\times 10^{7}$ hectares, accounting for approximately 35% of the total grain crop area. Its yield and quality are directly linked to national food security, livestock industry development, and the supply of industrial raw materials [1,2]. However, maize is continuously threatened by various pests throughout its growth cycle, with corn borer, army worm, cutworm, and other pests being particularly rampant [3]. According to Chinese statistics, annual maize yield losses due to pest damage average 8%–12%, reaching over 30% in severe outbreak years, resulting in direct economic losses exceeding tens of billions of RMB [4–6]. Traditional pest monitoring methods rely on visual inspection by plant protection experts or farmers’ field observations [7]. These approaches are not only time-consuming, labor-intensive, and limited in coverage but also highly subjective. Misjudgments often lead to indiscriminate pesticide application, exacerbating issues such as pesticide residues, pest resistance, and environmental pollution [8]. These challenges directly contradict the principles of sustainable agriculture and integrated pest management, which emphasize precise, eco-friendly, and economically viable control strategies. Consequently, the development of a pest detection approach that operates effectively under field conditions is of considerable research interest and practical relevance for supporting sustainable improvements in crop productivity.

With the rapid development of smart agriculture and precision farming, computer vision technology has been widely recognized as a core alternative to manual inspection and patrols [9]. Early research primarily focused on handcrafted low-level features such as color, texture, and shape, integrated with conventional classification approaches, including SVM and RF models [10], achieving classification accuracies of 80%–90% for single-pest images under controlled laboratory conditions [11]. However, natural field environments involve numerous uncertainties, including diverse pest postures and complex backgrounds, which significantly reduce the robustness and generalization capability of traditional methods [12]. In recent years, advances in deep learning—most notably within Convolutional Neural Network (CNN) frameworks—have exhibited substantial benefits in automated feature extraction and integrated end-to-end model optimization. These developments have introduced a refined methodological pathway for pest identification in highly variable agricultural environments [13,14]. Within the broader landscape of CNN-based detection models, YOLO (You Only Look Once), which follows a one-stage detection paradigm, has attained considerable research attention for real-time pest surveillance, primarily owing to its compact structural design, rapid inference characteristics, and its practicality for deployment on edge computing platforms [15,16].

Since Redmon et al. first proposed YOLO in 2016, the framework has undergone multiple iterations, including YOLOv2–YOLOv8 [17],YOLOv9 [18], YOLOX [19], YOLOv10 [20], and the latest YOLOv11 [21], with continuous improvements in detection accuracy and inference efficiency. In the agricultural domain, scholars have conducted numerous valuable explorations based on the YOLO series: Roy et al. [22] embedded DenseNet into the YOLOv4 backbone, proposing Dense-YOLOv4 for mango fruit detection in complex orchard environments, achieving an mAP@0.5 of 84.3%; Lawal et al. [23] improved the anchor mechanism and activation function of YOLOv3 to develop YOLO-Tomato, enabling real-time recognition of tomato early blight with a detection speed of 52 FPS; Zhang et al. [24] integrated spatial pyramid pooling with YOLOv3, proposing YOLOv3-SPP, which increased the recognition accuracy for small-sized rice pests to 88.07%; Tian et al. [25] optimized YOLOv3’s low-resolution feature layers using DenseNet, proposing YOLOv3-Dense, which improved the detection accuracy for apple anthracnose spots by 6.4%. Although these studies have achieved promising results in specific crop–disease/pest scenarios, directly applying existing YOLO models to maize pest detection still faces three common challenges:

(1) Large Morphological Variability: Maize pests exhibit significant morphological differences across growth stages, with substantial variations in body color and patterns within the same category. This leads to dispersed feature distributions, making it difficult for traditional convolutional kernels to fully capture discriminative information, thereby increasing the occurrence of erroneous detections and overlooked targets.
(2) Severe Cluttered Background Interference: In field-captured environments, pests often exhibit high similarity in color and texture to the background, introducing substantial redundant noise and making the model prone to false detections.
(3) High miss-detection rate of pests: For maize pests that are small in size, faint-edged, and leaf-colored, deep features lose localization detail due to overly low resolution, while shallow features lack semantic discrimination because of insufficient receptive field. When the two are simply summed, pest signals are drowned out by the background, causing miss rates of pests to remain high.

In response to the aforementioned limitations, a real-time maize pest detection model, termed WSD-YOLO, is developed by extending the latest YOLOv11n architecture with WMConv, SHLT, and DAWA modules. The main contributions of this study are outlined below:

(1) Construct a Windmill Convolution (WMConv) module to replace standard convolutions in the backbone network. This module employs four-directional asymmetric kernels (1×3, 3×1) for parallel feature extraction and expands the receptive field through an asymmetric padding strategy. It enhances the network’s sensitivity to pest textures in any direction with almost no increase in computational cost, significantly improving low-level feature representation, effectively suppressing complex background noise, and providing stronger adaptability to small and multi-scale pests.
(2) Design a Single-Scale High-Level Transformer (SHLT) module to replace the original SPPF in YOLOv11n. Its core idea is to perform self-attention only on the single layer with the richest semantics and lowest resolution, capturing long-range dependencies with minimal computational overhead to suppress complex background noise, thereby improving the recall rate for tiny pests.
(3) Design a Dual-Attention Weighted Aggregation (DAWA) module to enhance feature expression in the detection head. DAWA performs weighted fusion of features at the same scale across the three spatial resolutions (P3, P4 and P5) via channel-spatial dual-path cross-attention, enabling the detection head to simultaneously acquire high-contrast target information and rich texture details, significantly improving detection accuracy for small, medium, and large targets.

## 2. Materials

### 2.1 Dataset

This study adopts a dataset derived from the IP102 benchmark, a publicly accessible and large-scale collection of insect pest images [26–28]. IP102 has been widely recognized as a standard reference dataset within the domain of agricultural pest classification. It is extensive in scale, containing over 75,000 images and encompassing 102 pest categories that affect various crops such as rice, wheat, maize, and fruits [26]. Images contained in the IP102 dataset mainly originate from real-world field environments, thus presenting rich real-world challenges such as complex backgrounds and variations in pest morphology across different growth stages and viewing angles, granting it significant research value [29,30].

However, as a general pest dataset, IP102 suffers from highly imbalanced class distribution and covers multiple crop types. For the specific task of maize pest detection, the large number of non-maize pest categories within the dataset can introduce irrelevant feature interference, which is detrimental to achieving optimal model performance.

**Fig 1:**
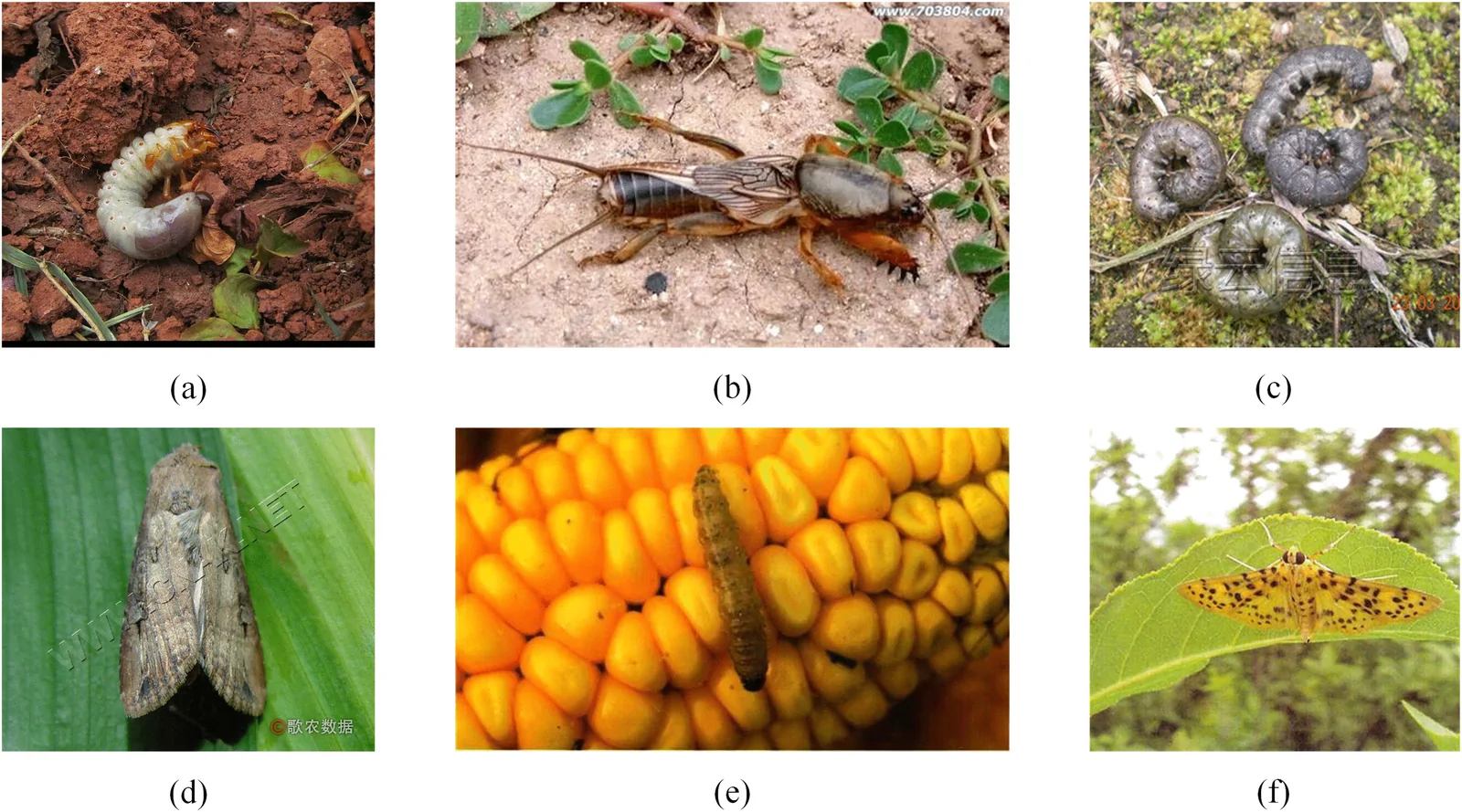
A selection of raw images from the IPMaize. **(a)** Sample(Grub). **(b)** Sample(Mole cricket). **(c)** Sample(White margined moth). **(d)** Sample(Black cutworm). **(e)** Larval sample(Peach borer). **(f)** Adult sample(Peach borer).

To address these issues and focus specifically on maize pest detection, a dedicated subset was constructed from the IP102 dataset by retaining only the pest species associated with maize. This subset comprises 13 classes with a total of 4,533 images, which we refer to as the IPMaize dataset in this paper. The selected categories correspond to maize-related pest species included in the IP102 dataset and were determined according to the official category definitions of IP102 and previous studies on maize pests [3–6]. All images and their corresponding bounding-box annotations were directly adopted from the officially released IP102 object detection dataset. After category extraction, all retained images and annotations were manually reviewed to verify the consistency among image content, category labels, and bounding-box annotations. Details of the selected categories are listed in Table 1, and Fig 1 presents representative samples.

**Table 1:** Pest species and sample numbers in IPMaize.

ID	Pest Species	Number
0	Grub	436
1	Mole cricket	868
2	Wireworm	424
3	White margined moth	49
4	Black cutworm	300
5	Large cutworm	153
6	Yellow cutworm	194
7	Red spider	160
8	Corn borer	425
9	Army worm	206
10	Aphids	874
11	Potosia brevitarsis	203
12	Peach borer	241
Total		4533

The IPMaize dataset was divided into training, validation, and testing subsets at a ratio of 8:1:1 using stratified sampling according to pest categories, ensuring that each subset preserved the original class distribution. The same partition was adopted for all experiments. No additional offline class balancing strategy, such as oversampling or synthetic sample generation, was applied, and the original class distribution was retained to evaluate the proposed model under realistic agricultural conditions. During model training, online data augmentation provided by the Ultralytics YOLO11 framework was employed, including Mosaic augmentation, HSV color transformation, random horizontal flipping, scaling, and translation. Since these augmentation operations were dynamically performed during training, they did not generate additional permanent images or change the original class distribution of the IPMaize dataset. Therefore, no fixed augmented dataset or class-wise sample statistics after augmentation exist.

### 2.2 Baseline model YOLOv11

**Fig 2:**
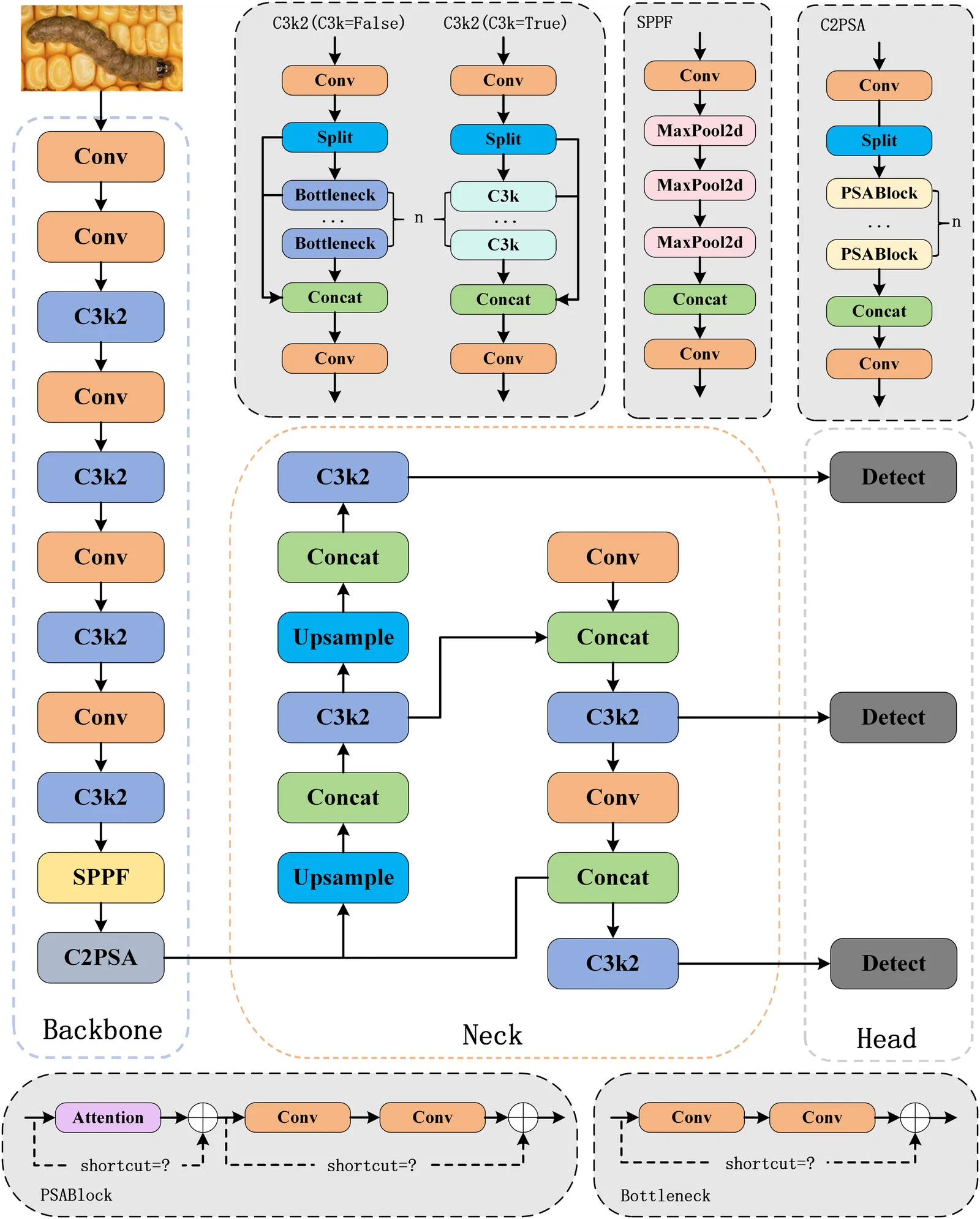
The YOLO11 architecture is composed of several key components, including the BottleNeck, C3k, C3k2, PAS Block, SPPF, and C2PSA modules.

As shown in Fig 2, YOLOv11 adopts a modular architecture consisting of a backbone, a neck, and a head. In this design, feature extraction is mainly handled by the backbone, which incorporates four functional modules, namely Conv, C3k2, fast Spatial Pyramid Pooling (SPPF), and C2PSA. The C3k2 module serves as a substitute for the C2f module employed in YOLOv8, featuring an architecture built around a C3k module. The incoming feature map is split into two parallel processing streams, with one branch following a lightweight forward propagation process, while the other is processed through stacked 3×3 bottleneck residuals before being concatenated and fused. This design not only reduces repetitive gradient computation but also enhances semantic abstraction by providing an equivalent larger receptive field [31,32]. The SPPF module functions as a multi-scale pooling component, effectively enlarging the multi-scale receptive field without increasing computational load. Following the SPPF module, the C2PSA module is incorporated. The C2PSA module utilizes PSA (Partial Spatial Attention) to operate on different branches of the feature map, which are then concatenated. This mechanism ensures the model focuses on spatial information, refining its ability to selectively attend to regions of interest while maintaining a balance between computational cost and detection accuracy [33,34].

The Neck module combines a Feature Pyramid Network (FPN) with a Path Aggregation Network (PAN) to enable the integration of feature representations across multiple hierarchical stages. The FPN establishes a top–down pathway that consolidates abstract semantic cues typically diminished in deeper layers of the architecture [35]. Complementing this, the PAN introduces a bottom–up information stream that strengthens the transmission of fine-grained spatial details toward higher-level layers, thereby enhancing the network’s capacity for precise localization and small-object recognition by sustaining high spatial resolution [36]. Through the collaborative interaction of the FPN’s descending path and the PAN’s ascending path, the Neck component achieves efficient fusion of features originating from diverse representational levels.

Within the detection head, depthwise separable convolutions are adopted in YOLOv11 to enable the separation of localization and classification branches. To address variations in object scale, the network outputs feature representations at three distinct resolution levels, allowing effective recognition of targets with diverse sizes. This architectural arrangement decreases parameter count and overall model complexity, while simultaneously improving the network’s generalization capability and robustness [37]. Moreover, redundant bounding boxes are removed through the application of Non-Maximum Suppression (NMS), which helps to refine the final detection results [38].

Under a strict budget of 2.58 M parameters and 6.3 GFLOPs (input 640×640), YOLOv11 achieves an effective balance between detection accuracy and computational cost, which supports its applicability in edge-device deployment scenarios. However, the performance of the YOLOv11 model still exhibits notable limitations. In particular, detection accuracy significantly degrades when the image background is complex, the distinction between the object and its surrounding background is relatively weak, or when target pests exhibit substantial morphological variations due to different developmental stages. These observations suggest that the current model’s robustness under complex scenarios, as well as its capability to capture variations in target shapes, still requires further improvement.

## 3. Methods

**Fig 3:**
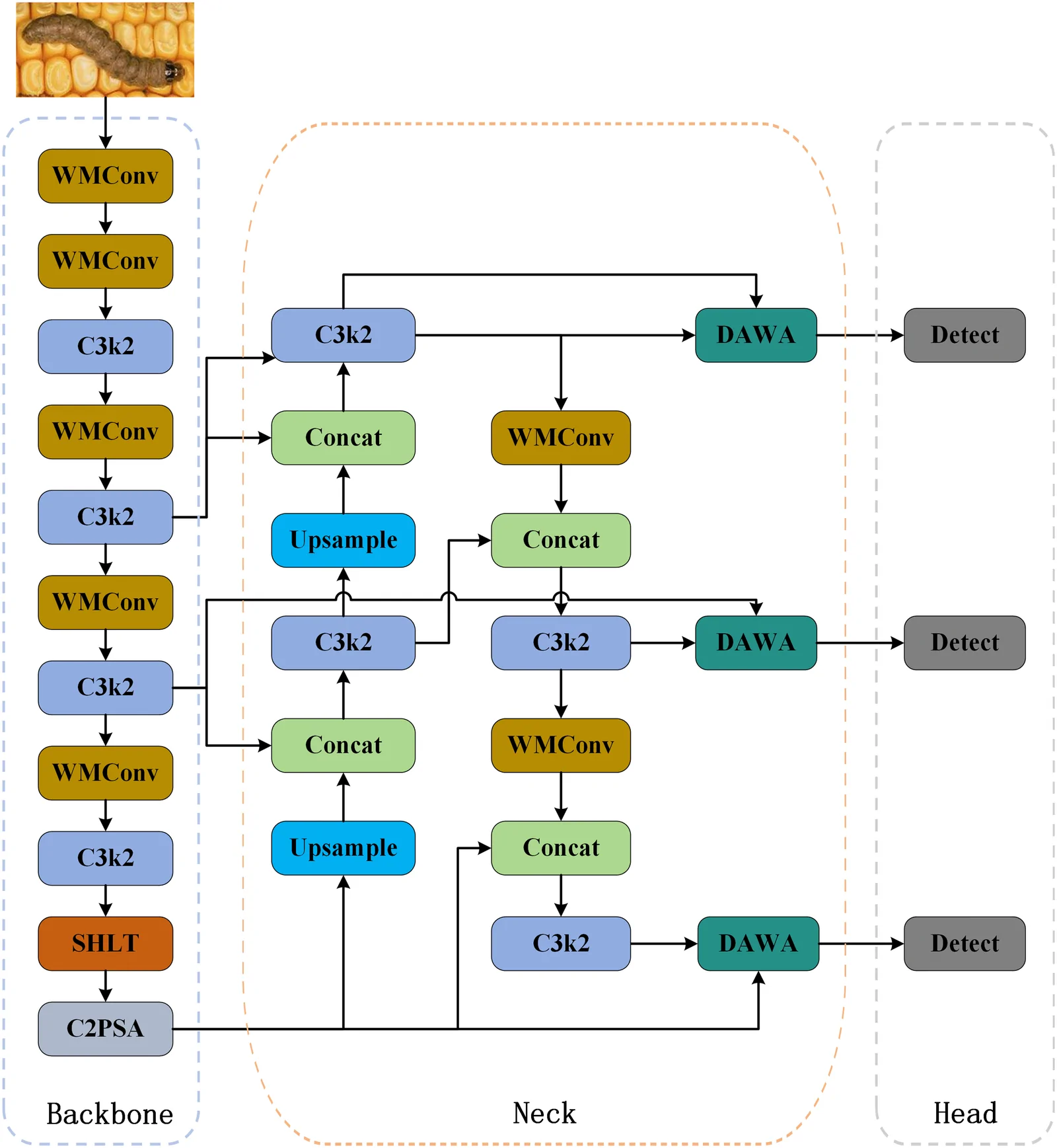
The architecture of WSD-YOLO.

To address the field imaging characteristics of maize pests, namely significant target morphological variation and cluttered backgrounds, this paper proposes WSD-YOLO. As shown in Fig 3, the model is built upon YOLOv11n by retaining its original PAFPN and decoupled head topology, while implementing three key upgrades to critical feature extraction and fusion nodes.

Fig 3 presents an overview of the WSD-YOLO architectural design:

(1) WMConv replaces the standard Conv modules in the Backbone and the downsampling layers in the Neck. This enhancement increases sensitivity to textures in arbitrary directions, significantly strengthening the expressive capability of low-level features.
(2) SHLT substitutes the SPPF module. By applying attention solely to high-level semantic features, it injects global contextual information at a relatively low computational cost, achieving efficient global self-attention modeling.
(3) DAWA is embedded prior to the detection heads. It performs cross-branch weighted fusion of features at the same scale, further boosting the recall rate for morphologically diverse pests and significantly improving detection accuracy for small, medium, and large targets.

All three improvements preserve the original gradient paths and output channel dimensions. Consequently, they allow for partial weight compatibility with the standard YOLOv11n, facilitating subsequent deployment on edge devices and iterative optimization.

### 3.1 WindmillConv (WMConv)

**Fig 4:**
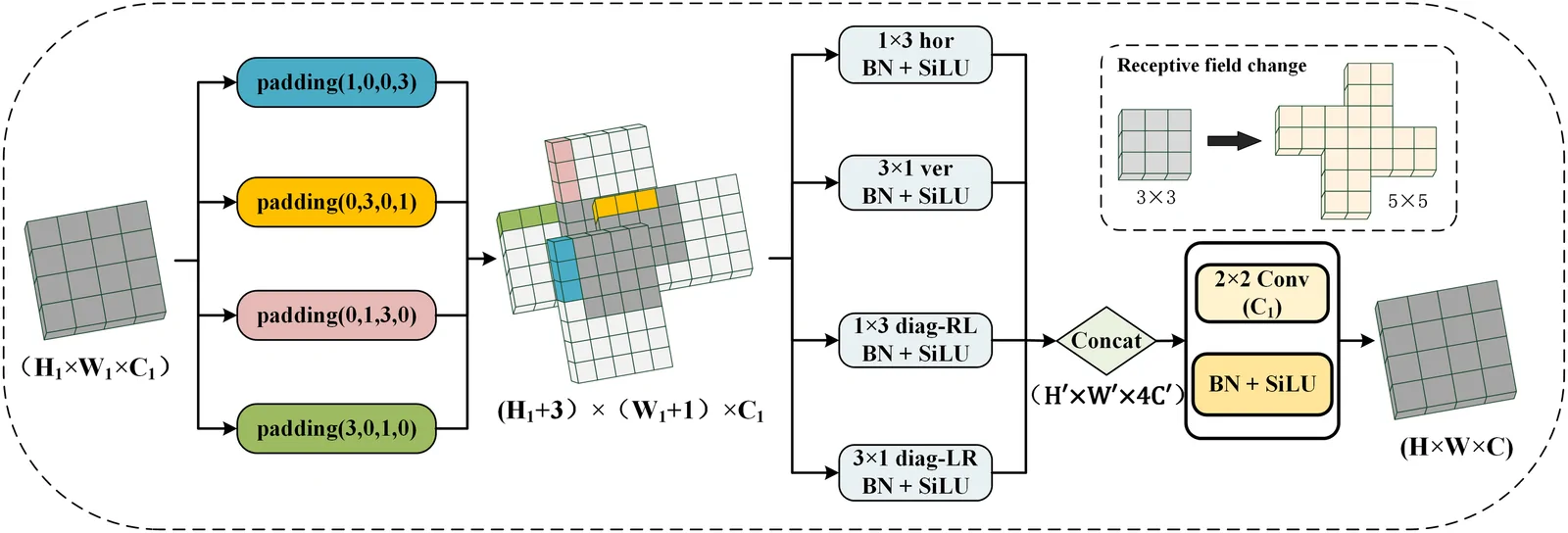
The network architecture of WMConv.

Maize pests in field images often exhibit characteristics such as arbitrary posture, slender edges, and randomly oriented textures. The isotropic sampling grid of the standard 3×3 square convolutional kernel is unable to provide differentiated responses to textures of arbitrary directions. Through its four-directional asymmetric kernels and variable-phase padding, WMConv expands the effective receptive field by 177% without increasing the parameter budget. Simultaneously, its directional convolutional kernels and channel compression mechanism can effectively capture the fine-grained textural features of maize pests (e.g., wing venation, antennae, and body segment edges). Fig 4 depicts the structural design of the WMConv network. It demonstrates superior texture discriminative power on the IPMaize dataset, which features complex backgrounds and small inter-class variations. The specific implementation is as follows:

(1) Four branches of 1×3 and 3×1 convolutions are respectively paired with asymmetric padding schemes such as (1,0,0,3) and (0,3,0,1) to form a “windmill”-shaped sampling grid.
(2) The output channels are split into four equal parts, concatenated, and then compressed via a 2×2 group convolution. This maintains directional diversity while controlling computational overhead.
(3) The WMConv module is replacing all original 3×3 Conv modules while keeping the overall network architecture unchanged.

The padding strategy is denoted as padding (left, right, top, bottom) and the symbols *H*_1_, *W*_1_, *C*_1_ correspond to the spatial dimensions and channel count of the input feature map *X*_1_. First, asymmetric padding is employed to construct horizontal and vertical convolution kernels across different image regions. To promote stable and efficient training, convolutional layers are complemented with Batch Normalization and SiLU-based nonlinear activation. WMConv performs parallel convolutions as described in the following formulas:


$$\begin{array}{c} X_{hor}=SiLU(BN({Conv}_{1\times 3\times C_{1},\;p=(1,0,0,3)}(X_{1}))) \end{array}$$ (1)

$$\begin{array}{c} X_{ver}=\mathrm{SiLU}(\mathrm{BN}({\mathrm{Conv}}_{3\times 1\times C_{1},p=(0,3,0,1)}(X_{1}))) \end{array}$$ (2)

$$\begin{array}{c} X_{\text{diag-RL}}=\text{SiLU}(\text{BN}({\text{Conv}}_{1\times 3\times C_{1},p=(0,1,3,0)}(X_{1}))) \end{array}$$ (3)

$$\begin{array}{c} X_{\text{diag-LR}}=\text{SiLU}(\text{BN}({\text{Conv}}_{3\times 1\times C_{1},p=(3,0,1,0)}(X_{1}))) \end{array}$$ (4)

$$\begin{array}{c} X^{\prime }=Concat(X_{hor},X_{ver},X_{diag\text{-}RL},X_{diag\text{-}LR}) \end{array}$$ (5)

where *X*_hor_, *X*_ver_, $X_{\text{diag}-\text{RL}}$, and $X_{\text{diag}-\text{LR}}$ represent the features captured by the horizontal, vertical, right-up-to-left-down diagonal, and left-up-to-right-down diagonal kernels, respectively. $X^{\prime }$ denotes the intermediate feature map after concatenating the outputs of the four-path windmill kernels, where $H^{\prime }$, $W^{\prime }$ denote the spatial dimensions of the intermediate feature map $X^{\prime }$ and its channel depth is $4C^{\prime }$ (since $C^{\prime }$ is the output channels of each branch):


$$\begin{array}{c} C^{\prime }=C_{1}/4 \end{array}$$ (6)

$$\begin{array}{c} H^{\prime }=\frac{H_{1}}{s}+1 \end{array}$$ (7)

$$\begin{array}{c} W^{\prime }=\frac{W_{1}}{s}+1 \end{array}$$ (8)

where *s* is the convolution stride to enable the plug-and-play deployment of the WMConv module and ensure its compatibility with the engineering design of convolutional neural networks (CNNs), the number of output channels in the 2×2 convolution layer is set to *C*_1_, which is identical to the number of input channels. The final output feature map is *X*, here, *H*, *W*, and *C* denote the feature map’s height, width, and channel count, respectively:


$$\begin{array}{c} X=\text{SiLU}(\text{BN}({\text{Conv}}_{2\times 2}(X^{\prime }))) \end{array}$$ (9)

$$\begin{array}{c} H=H^{\prime }-1 \end{array}$$ (10)

$$\begin{array}{c} W=W^{\prime }-1 \end{array}$$ (11)

the formula for computing the number of parameters of the convolution layer is:


$$\begin{array}{c} {\text{Conv}}_{\text{params}}=C_{1}\times C\times k,\;(\text{bias}=\text{False}) \end{array}$$ (12)

if *C*_1_ = *C*, the parameters of the 3×3 convolution and WMConv are calculated as follows:


$$\begin{array}{c} 3\times 3{Conv}_{params}=9C_{1}\times C=9C^{2} \end{array}$$ (13)

$$\begin{array}{c} {WMConv}_{\text{params}}=4\times \left(\frac{C_{1}}{4}\times C\times 3\times 1\right)+4C_{1}C=7C_{1}C=7C^{2} \end{array}$$ (14)

by employing four groups of parallel asymmetric kernels for feature extraction, WMConv reduces the parameter count of an individual convolution operator by approximately 22% compared with a standard 3×3 convolution, while expanding the theoretical receptive field from 3×3 to 5×5. As shown in the upper right corner of Fig 4, WMConv expands the effective receptive field by approximately 177% without increasing the parameter cost of the convolution operator.

To quantitatively illustrate the receptive field enlargement introduced by WMConv, the theoretical receptive field area is calculated as:


$$\begin{array}{c} \eta =\frac{A_{\text{WMConv}}-A_{\text{Conv}}}{A_{\text{Conv}}}\times 100\% \end{array}$$ (15)

where *A*_Conv_ denotes the theoretical receptive field area of standard convolution, and *A*_WMConv_ represents the theoretical receptive field area of the proposed WMConv module; η refers to the growth rate of receptive field area.

The effective receptive field expands from approximately 3×3 to 5×5, the receptive field area increases from 9 to 25, resulting in:


$$\begin{array}{c} \eta =\frac{25-9}{9}\times 100\%\approx 177\% \end{array}$$ (16)

**Fig 5:**
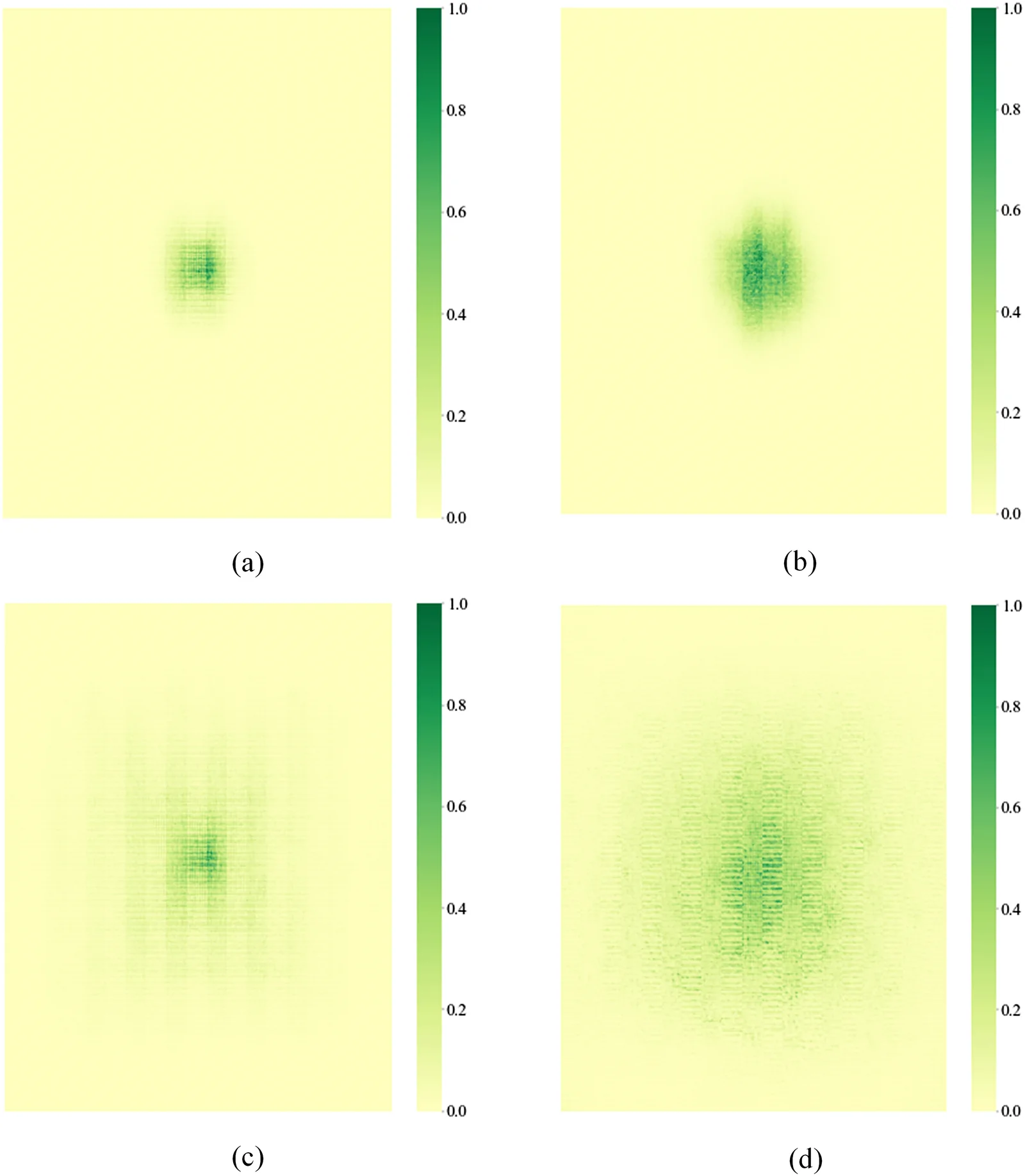
Effective receptive field (ERF) visualization of the baseline YOLOv11n and the proposed WSD-YOLO. **(a)** YOLOv11n (Layer 7). **(b)** WMConv (Layer 7). **(c)** YOLOv11n (Layer 22). **(d)** WMConv (Layer 22).

To further verify that the enlarged receptive field is not merely a theoretical derivation, the Effective Receptive Field (ERF) was visualized using the gradient-based ERF analysis method. Fig 5 compares the ERF distributions of the baseline YOLOv11n and the proposed WSD-YOLO at both the shallow feature extraction stage (Layer 7) and the deep feature extraction stage (Layer 22).

As shown in Fig 5(a) and Fig 5(b), compared with the baseline YOLOv11n, WSD-YOLO exhibits a noticeably broader activation region while maintaining strong responses around the target center in the shallow feature layer, indicating enhanced local structural perception. Furthermore, Fig 5(c) and Fig 5(d) show that the effective receptive field of the deep feature layer is also significantly enlarged, enabling richer contextual information to participate in feature extraction. These observations are consistent with the theoretical analysis presented above and qualitatively demonstrate that the proposed WMConv effectively enlarges the effective receptive field in the trained network.

WMConv is designed to strengthen multi-directional structural perception for arbitrarily oriented maize pests while maintaining lightweight computation. Representative convolution enhancement methods mainly improve feature extraction through different receptive-field modeling strategies. The Asymmetric Convolution Block (ACB) proposed in the Asymmetric Convolutional Network (ACNet) enhances kernel representation by combining horizontal and vertical asymmetric branches [39], whereas Wavelet Transform Convolution (WTConv) enlarges the effective receptive field through wavelet-domain frequency decomposition [40]. Different from these methods, WMConv constructs a windmill-shaped sampling pattern using four complementary asymmetric kernels with phase-shifted padding, enabling simultaneous modeling of horizontal, vertical, and diagonal texture responses within a unified lightweight operator. This design is particularly suitable for capturing arbitrary-oriented pest structures and fine-grained edge details in complex field environments.

### 3.2 Single-Scale High-Level Transformer (SHLT)

**Fig 6:**
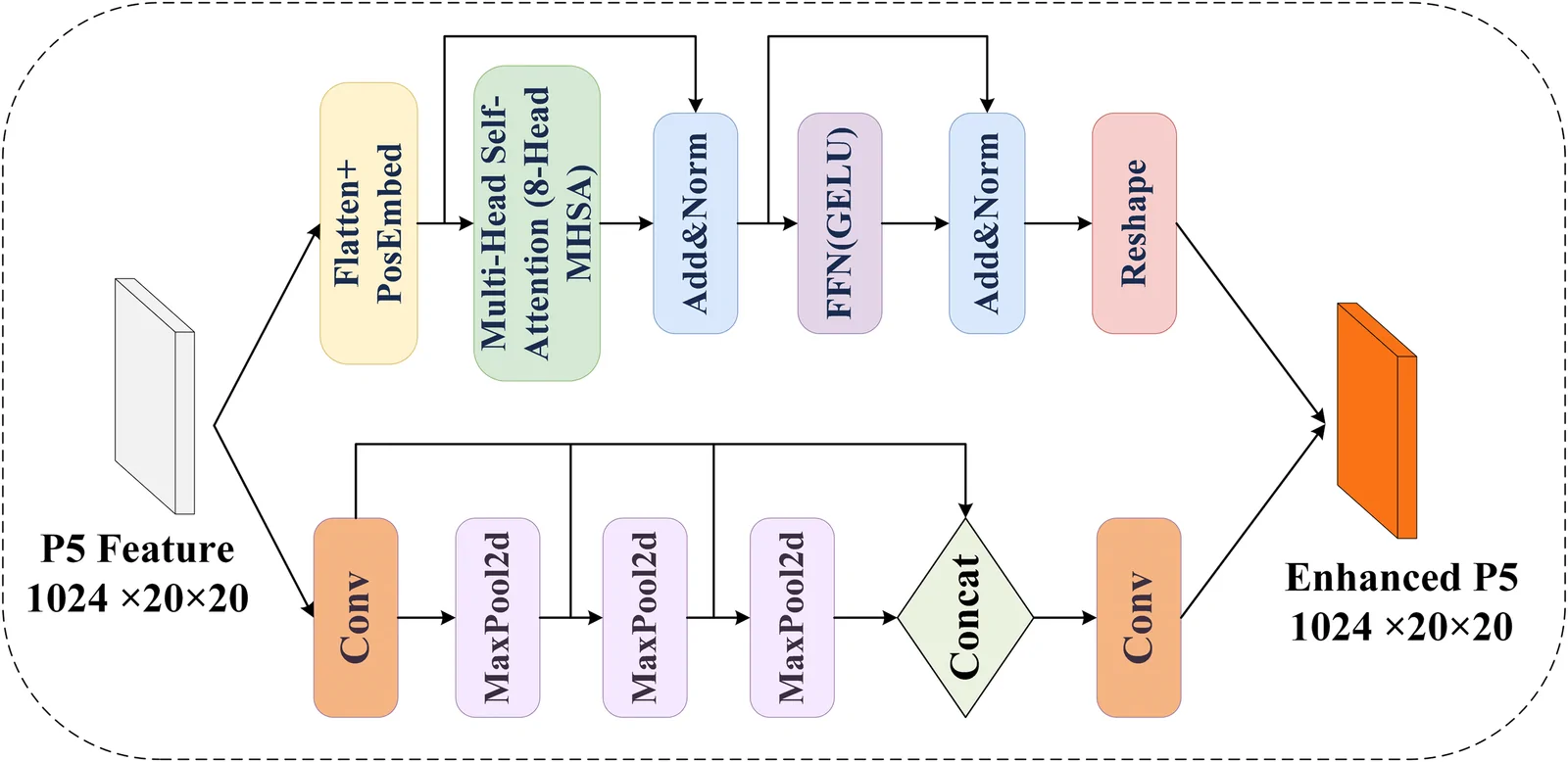
Comparative diagram of the network architectures of SPPF and SHLT. The upper branch depicts the SHLT architecture; the lower branch illustrates the network architecture of SPPF.

In field environments, maize pests often exhibit characteristics such as color similarity between the target and leaves, and abundant repetitive textures. The SPPF module obtains different receptive fields through cascaded max pooling while being computationally efficient, it merely concatenates local contexts and lacks the ability to model global dependencies. The feature processing of SPPF is illustrated by the lower branch in Fig 6.

This paper introduces the SHLT module to replace the original SPPF architecture in YOLOv11n. Its core concept is to apply self-attention solely to the single feature layer with the richest semantics and lowest resolution, because the semantically richest hierarchy captures sufficient global context, rendering multi-scale modeling unnecessary. This enables the capture of long-range dependencies at a minimal computational cost, enhancing the salience of pest regions while suppressing complex backgrounds, as demonstrated by the upper branch in Fig 6.

The input features are selected exclusively from the deepest layer of the backbone, the P5 feature map $X^{\prime }\in {\mathbb{R}}^{C\times H\times W}$ (where *C* = 1024, *H* = *W* = 20). This layer possesses the richest semantic information but the lowest spatial resolution, adhering to the single-scale design principle.

First, the two-dimensional feature map is transformed into a one-dimensional token sequence to prepare for subsequent Transformer operations.


$$\begin{array}{c} X_{\text{encoded}}=\text{Flatten}(X^{\prime })+P^{⊤}\in {\mathbb{R}}^{C\times N} \end{array}$$ (17)

where *X*_encoded_ represents the features after serialization and position encoding, where the complete formulation for Position Embedding (PosEmbed) is as follows:


$$\begin{array}{c} {\text{PE}}_{\left(\text{pos},2i\right)}=\sin\left(\frac{\text{pos}}{10000^{2i/C}}\right) \end{array}$$ (18)

For the position index of the sequence, denoted as pos∈[0,N−1], and the dimension index $i\in [0,\frac{C}{2}-1]$, the position-encoding matrix $\text{P}\in {\mathbb{R}}^{N\times C}$ can be expressed as:


$$\begin{array}{c} P=\left[\begin{array}{cccc} {PE}_{\left(0,0\right)} & {PE}_{\left(0,1\right)} & \cdots & {PE}_{\left(0,1023\right)}\\ {PE}_{\left(1,0\right)} & {PE}_{\left(1,1\right)} & \cdots & {PE}_{\left(1,1023\right)}\\ \vdots & \vdots & \ddots & \vdots \\ {PE}_{\left(399,0\right)} & {PE}_{\left(399,1\right)} & \cdots & {PE}_{\left(399,1023\right)} \end{array}\right] \end{array}$$ (19)

where N=H×W=20×20=400 represents the sequence length. The query, key, and value representations are obtained via linear projection as follows:


$$\begin{array}{c} Q=X_{\text{encoded}}^{⊤}W^{Q} \end{array}$$ (20)

$$\begin{array}{c} K=X_{\text{encoded}}^{⊤}W^{K} \end{array}$$ (21)

$$\begin{array}{c} V=X_{\text{encoded}}^{⊤}W^{V} \end{array}$$ (22)

where $W^{Q},W^{K},W^{V}\in {\mathbb{R}}^{C\times d_{k}}$ are learnable projection matrices, with $d_{k}$ denoting the projection dimension. Subsequently, long-range contextual relationships across the feature space are captured using a multi-head self-attention (MHSA) module. For the *i*-th attention head:


$$\begin{array}{c} {\mathrm{Attention}}_{i}=\mathrm{Softmax}\left(\frac{Q_{i}K_{i}^{⊤}}{\sqrt{d_{k}}}\right)V_{i} \end{array}$$ (23)

where $Q_{i},K_{i},V_{i}\in {\mathbb{R}}^{d_{k}\times N}$ denote the feature projections associated with the *i*-th attention head. Subsequently, representations from multiple attention heads are integrated and further processed using a linear transformation:


$$\begin{array}{c} Z=\mathrm{Concat}({\text{Attention}}_{1},...,{\text{Attention}}_{n})W^{O} \end{array}$$ (24)

where *Z* is the output of the multi-head self-attention mechanism, with $W^{O}\in {\mathbb{R}}^{h\cdot d_{k}\times C}$ being the output projection matrix and *h* the number of attention heads. Subsequently, a residual connection is applied followed by layer normalization to stabilize the training process:


$$\begin{array}{c} Z^{\prime }=LayerNorm(Z+X_{\text{encoded}}) \end{array}$$ (25)

where $Z^{\prime }$ is the enhanced feature obtained after stable training, where the specific computation of layer normalization is as follows:


$$\begin{array}{c} Z^{\prime }=\gamma \cdot \frac{(Z+X_{\text{encoded}})-\mu }{\sqrt{\sigma^{2}+\varepsilon }}+\beta \end{array}$$ (26)

where μ and $\sigma^{2}$ are the mean and variance, respectively, and γ and β are learnable parameters. This is followed by the Feed-Forward Network (FFN) with GELU activation:


$$\begin{array}{c} X^{\prime \prime }=FFN(Z^{\prime })={Linear}_{c}(GELU({Linear}_{dff}(Z^{\prime }))) \end{array}$$ (27)

where $X^{\prime \prime }$ represents the output of the Feed-Forward Neural Network (FFN), which is composed of two linear transformation layers with a GELU activation function applied between them. The hidden layer dimension dff is typically set to four times the input dimension. This is followed by a second residual connection and layer normalization:


$$\begin{array}{c} Z^{\prime \prime }=LayerNorm(X^{\prime \prime }+Z^{\prime }) \end{array}$$ (28)

where $Z^{\prime \prime }$ represents the final result after the second residual connection and layer normalization. Finally, the processed sequence is transformed back into a feature map format, yielding the reshaped feature map *X*:


$$\begin{array}{c} X=\varphi (Z^{\prime \prime })\in {\mathbb{R}}^{C\times H\times W} \end{array}$$ (29)

where φ denotes the spatial dimension reshaping operation. The final features retain the original spatial resolution while integrating global contextual information. This integration can effectively suppress the noise from repetitive leaf textures and enhance the subsequent detection head’s response to weak-contrast regions, such as pest edges and spots.

SHLT is proposed to establish efficient long-range contextual modeling by applying global self-attention only to the highest semantic feature map. Representative Transformer-based feature enhancement methods mainly improve contextual representation through different attention mechanisms. Large Separable Kernel Attention (LSKA) captures long-range spatial dependencies by employing large separable kernels [41], whereas Multi-cognitive Visual Adapter (Mona) enhances feature representation through lightweight visual adaptation [42]. Different from these approaches, SHLT performs global self-attention only on the deepest semantic feature map. This single-scale design preserves sufficient global contextual information while avoiding redundant attention computation on high-resolution feature maps, resulting in a better balance between detection accuracy and computational efficiency.

### 3.3 Dual-Attention Weighted Aggregation (DAWA)

**Fig 7:**
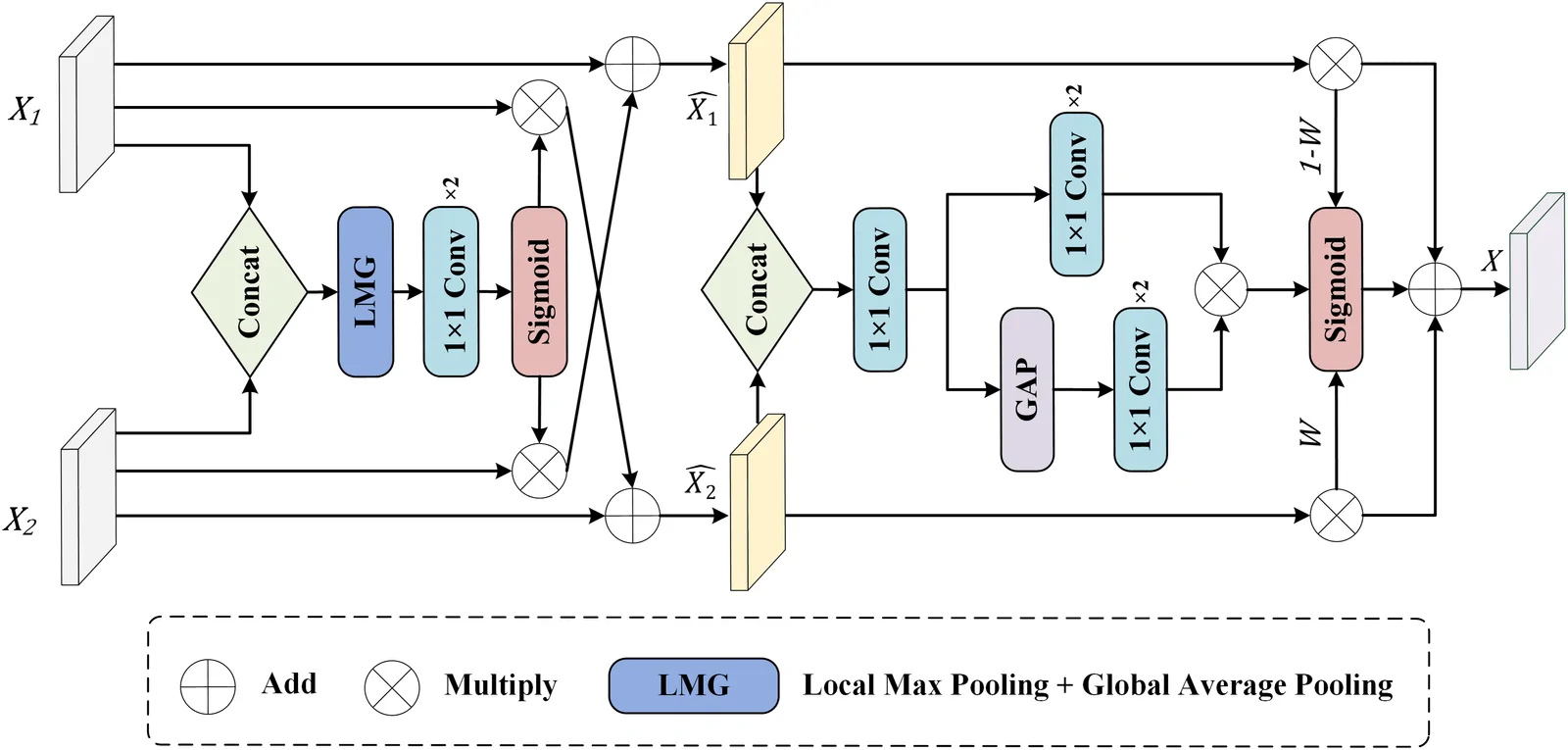
The network architecture of DAWA.

To address the imbalance between target and background weights within same-scale features, which leads to high miss rates for morphologically diverse maize pests, this paper proposes DAWA (Dual-Attention Weighted Aggregation). Fig 7 provides a depiction of the architectural design of the DAWA network.

It takes two feature maps from the same scale as input: *X*_1_ (from the Backbone layer, rich in texture details) and *X*_2_(from the Neck layer, strong in semantic information). LMG (Local-Max and Global-Avg Pooling) combines local maximum and global average pooling, enabling the capture of both local image details and holistic global context. This provides richer feature representations, which helps the model not only enhance target features but also effectively handle complex backgrounds, thereby improving model robustness and generalization. Additionally, it supplies more informative features for subsequent attention mechanisms. It then achieves detail enhancement in complex backgrounds through Dual-Path Channel-Spatial Attention and Adaptive Weighted Fusion.

The $X_{1}\in {\mathbb{R}}^{C\times H\times W}$ represents the shallow features from the Backbone (rich in texture details), and $X_{2}\in {\mathbb{R}}^{C\times H\times W}$ represents the deep features from the Neck (strong in semantics). They are input for subsequent weight generation. The feature enhancement process is defined as follows:


$$\begin{array}{c} \hat{X_{1}}=X_{1}⊕(X_{2}⊗\delta (1\times 1{Conv}^{2}(LMG(Concat(X_{1},X_{2}))))) \end{array}$$ (30)

$$\begin{array}{c} \hat{X_{2}}=X_{2}⊕(X_{1}⊗\delta (1\times 1{Conv}^{2}(LMG(Concat(X_{1},X_{2}))))) \end{array}$$ (31)

where $\hat{X_{1}}$ and $\hat{X_{2}}$ represent the enhanced features generated after feature reinforcement, here, ⊕ represents addition performed on corresponding elements, ⊗ indicates element-wise multiplication, and Concat signifies the operation of merging features along the channel axis. δ and LMG refer to the Sigmoid activation function and Local-Max and Global-Avg Pooling, respectively. The LMG layer fuses the results of local max pooling and global average pooling. $1\times 1{Conv}^{2}$ indicates two cascaded pointwise convolutional layers. The enhanced features are aggregated in the channel dimension before being processed by parallel attention mechanisms for fusion weight generation. The fusion weight generation process can be expressed as:


$$\begin{array}{c} A_{c}=1\times 1{Conv}^{2}(GAP(1\times 1Conv(Concat(\hat{X_{1}},\hat{X_{2}})))) \end{array}$$ (32)

$$\begin{array}{c} A_{s}=1\times 1{Conv}^{2}(1\times 1Conv(Concat(\hat{X_{1}},\hat{X_{2}}))) \end{array}$$ (33)

$$\begin{array}{c} W=Sigmoid(A_{c}⊗A_{s}) \end{array}$$ (34)

where $A_{c}$ and $A_{s}$ represent the weights corresponding to channel-wise and spatial attention, respectively, and *W* denotes the fusion weight. *GAP* stands for Global Average Pooling. Since features extracted at early stages and those derived from deeper layers are complementary, we generate the fusion weight *W* based on $\hat{X_{1}}$ and apply its complementary weight 1−W to $\hat{X_{2}}$. Accordingly, the resulting feature integration procedure can be formulated as follows:


$$\begin{array}{c} X=(W⊗\hat{X_{1}})⊕((1-W)⊗\hat{X_{2}}) \end{array}$$ (35)

DAWA is designed to adaptively aggregate complementary backbone and neck features at the same spatial scale before prediction. Representative attention-based feature fusion methods mainly improve feature interaction through different attention strategies. The Convolution and Attention Fusion Module (CAFM) jointly models local and global information by integrating convolution and attention branches [43], whereas Content-Guided Attention Fusion (CGAFusion) generates adaptive attention weights according to feature content [44]. Different from these approaches, DAWA performs adaptive complementary aggregation of backbone and neck features through channel-spatial dual attention. This design generates more discriminative feature representations before prediction while maintaining a lightweight architecture.

### 3.4 Design rationale and comparison with existing methods

To further clarify the novelty of the proposed modules, Table 2 summarizes the fundamental differences between WMConv, SHLT, and DAWA and representative feature enhancement methods from the perspectives of design objective, information modeling strategy, and architectural characteristics. As can be seen, although these representative methods improve feature representation through different mechanisms, the proposed modules are designed specifically for maize pest detection under complex field environments and therefore differ fundamentally in both design motivation and implementation strategy.

**Table 2:** Fundamental differences between the proposed modules and representative feature enhancement methods.

Proposed Module	Representative Method	Design Objective of Representative Method	Fundamental Difference of Proposed Module
WMConv	WTConv	Expand receptive field through wavelet decomposition	Spatial multi-directional structural perception using windmill-shaped asymmetric kernels rather than frequency-domain decomposition
WMConv	ACB (ACNet)	Strengthen kernel representation using asymmetric branches	Four-orientation complementary directional sampling for arbitrary-oriented pest texture modeling
SHLT	LSKA	Capture long-range dependencies via large separable kernels	Single-scale transformer-based contextual modeling on the deepest semantic feature map
SHLT	Mona	Lightweight visual adaptation for feature enhancement	Integrated contextual enhancement within the detector architecture rather than adapter-based feature adaptation
DAWA	CGAFusion	Attention-guided feature interaction	Adaptive same-scale complementary aggregation between backbone and neck features
DAWA	CAFM	Joint convolution-attention feature fusion	Detection-oriented dual-attention aggregation rather than independent channel-spatial recalibration

## 4. Experimental results and discussion

### 4.1 Experimental environment and parameter settings

The experiments were conducted using the Windows 10 Professional operating system, version 22H2. The CPU was an Intel Core i9-12900K @ 3.2 GHz with 128 GB of RAM. The GPU configuration consisted of two NVIDIA GeForce RTX 3090 cards, each with 24 GB of memory. The software environment included CUDA version 12.1, PyTorch version 2.2.2, and Python version 3.10. The detailed experimental configuration is shown in Table 3.

**Table 3:** Experimental setup and environment.

Configuration	Parameter
Operating system	Windows 10
CPU	Intel Core i9-12900K@3.2GHz
GPU	NVIDIA GeForce RTX 3090
RAM	128G
Languages	Python 3.10
Framework	PyTorch 2.2.2
Operating platform	CUDA 12.1

Proper configuration of hyperparameters is essential for stable model training and fair performance comparison, and the detailed settings are presented in Table 4. Considering the pronounced domain differences between the IPMaize dataset and generic object-detection datasets, pretrained weights may introduce feature bias unrelated to maize pests. Therefore, all models were trained from scratch under identical settings to ensure that the observed performance differences were primarily attributable to their architectural designs.

**Table 4:** Hyperparameters for model training.

Name	Value
lr0	0.01
lrf	0.01
Optimizer	SGD
Momentum	0.937
Batch	32
imgsz	640
IoU	0.7
epochs	300
Random seeds	42,2026,3407

All input images were resized to 640×640 pixels, which preserves sufficient spatial details for small pest targets while maintaining manageable computational cost. The batch size was set to 32 to ensure stable gradient estimation and efficient utilization of the available GPU memory. SGD was selected as the optimizer because it provided stable convergence during preliminary training, with a momentum of 0.937 to accelerate optimization and reduce gradient oscillation. The initial learning rate (lr0) was set to 0.01, while the final learning-rate factor (lrf) was set to 0.01, resulting in a final learning rate of approximately $1.0\times 10^{-4}$.

An IoU threshold of 0.7 was adopted to provide a relatively strict matching criterion between predicted and ground-truth bounding boxes. Since the models were trained from randomly initialized weights, the maximum number of training epochs was set to 300 to allow sufficient convergence. Meanwhile, early stopping with a patience of 100 epochs was employed to terminate training when the validation performance no longer improved, thereby avoiding unnecessary computation and potential overfitting. The same hyperparameter settings were applied to all compared models to ensure experimental fairness.

To improve the reproducibility of the experimental results, three random seeds (42, 2026, and 3407) were selected for repeatability analysis.To ensure experimental reproducibility, we performed repeated experiments using three independent random seeds (42, 2026, and 3407) on the proposed model, baseline model and ablation studies to further validate the reliability of performance improvements. The repeated results were reported as mean ± standard deviation where applicable. For all other main experiments, random seed 42 was uniformly adopted to guarantee consistent experimental conditions across different detection frameworks. Since YOLOv11n and WSD-YOLO were evaluated under identical random seeds, a two-tailed paired Student’s t-test was employed to evaluate whether the observed performance differences were consistent across repeated experiments. A p-value smaller than 0.05 was considered indicative of statistically significant differences.

### 4.2 Performance evaluation indicators

To achieve a thorough and unbiased assessment of the proposed object detection approach, several evaluation indicators were employed,including Precision (P), Recall (R), F1-score, mean Average Precision (mAP) and frames per second (FPS). Their formulations are given in Equations (36)–(40).


$$\begin{array}{c} Precision(P)=\frac{TP}{TP+FP} \end{array}$$ (36)

$$\begin{array}{c} Recall(R)=\frac{TP}{TP+FN} \end{array}$$ (37)

$$\begin{array}{c} AP_{i}=\int_{0}^{1}P_{i}(R_{i})\;dR_{i} \end{array}$$ (38)

$$\begin{array}{c} F_{1}=2\cdot \frac{P\cdot R}{P+R} \end{array}$$ (39)

$$\begin{array}{c} \text{mAP}=\frac{1}{C}\sum_{i=1}^{C}{\text{AP}}_{i} \end{array}$$ (40)

where Precision (P) quantifies the ratio of positive instances that are correctly identified by the model relative to all outputs labeled as positive, thereby indicating the trustworthiness of positive predictions. Recall (R) is described as the fraction of true positive samples that are successfully recognized among all annotated positive instances, which reflects the model’s ability to cover the target category. The F1-score is calculated as the harmonic mean of Precision and Recall, serving as a balanced metric that jointly reflects both measures. Mean Average Precision (mAP) comprehensively considers the performance of P and R under varying thresholds, functioning as a primary indicator for measuring the detector’s global recognition capability. This value is computed by combining the areas enclosed by the precision–recall curves across all object classes, serving as a comprehensive indicator of model stability and robustness in multi-class scenarios. In this study, a True Positive (TP) denotes a pest instance that is correctly recognized by the model, whereas a False Positive (FP) represents a non-pest region, such as background or other categories, that is incorrectly classified as a pest. A False Negative (FN) refers to a pest instance that exists in the image but is not correctly identified by the model. Here *C* denotes the number of pest categories under consideration, and $P_{i}$ and $R_{i}$ denote the precision and recall metrics of the *i*-th pest category, respectively. Frames per second (FPS) denotes the number of images that can be processed and detected by the network per second, which is adopted as the speed metric for object detection. Inference speed was evaluated using single-image inference (batch size = 1) on a single NVIDIA GeForce RTX 3090 GPU with an input imgsz of 640×640. To facilitate fair comparison with previous studies, the average inference latency (ms/image) is also reported.

### 4.3 Detection results of WSD-YOLO

**Fig 8:**
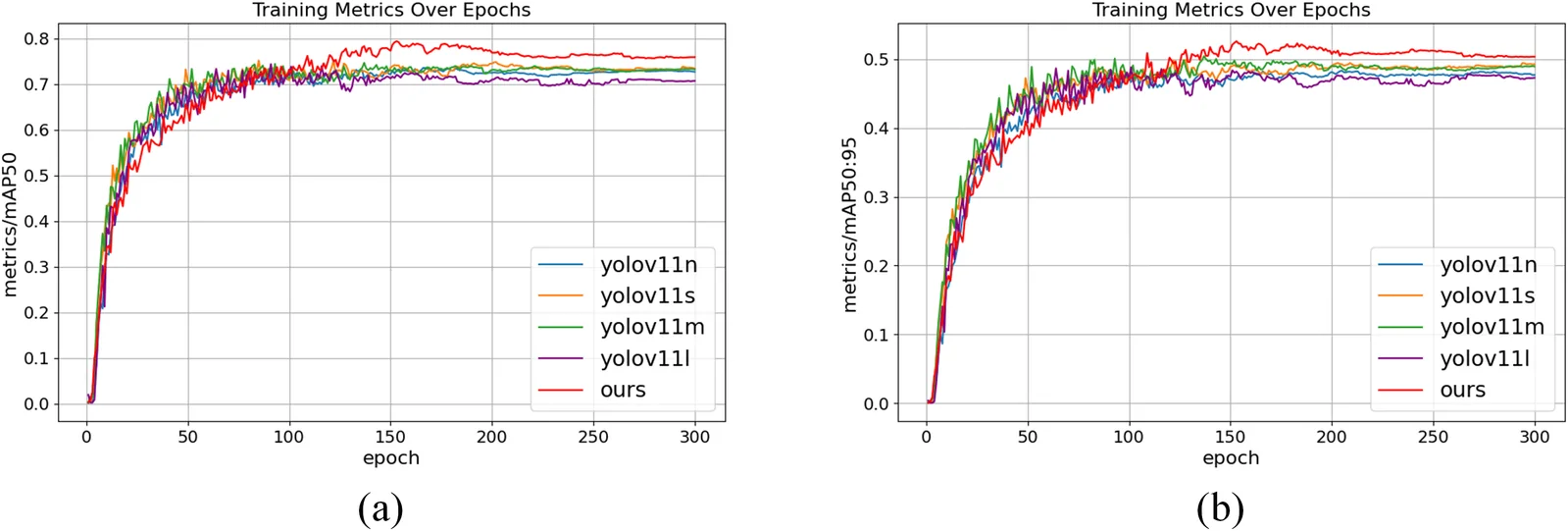
Performance comparison curves of WSD-YOLO and the YOLOv11 series. **(a)** mAP@0.5. **(b)** mAP@0.5:0.95.

In this experiment, we compared our method with the baseline models of the YOLOv11 series on the IPMaize dataset, as illustrated in Fig 8.

The figure presents the complete convergence process of WSD-YOLO on this dataset. It can be clearly observed that the integration of the WMConv, SHLT, and DAWA modules demonstrated a comparatively strong positive performance. Notably, our model achieved significantly higher scores in both mAP@0.5 and mAP@0.5:0.95 compared to the YOLOv11 series models.

**Fig 9:**
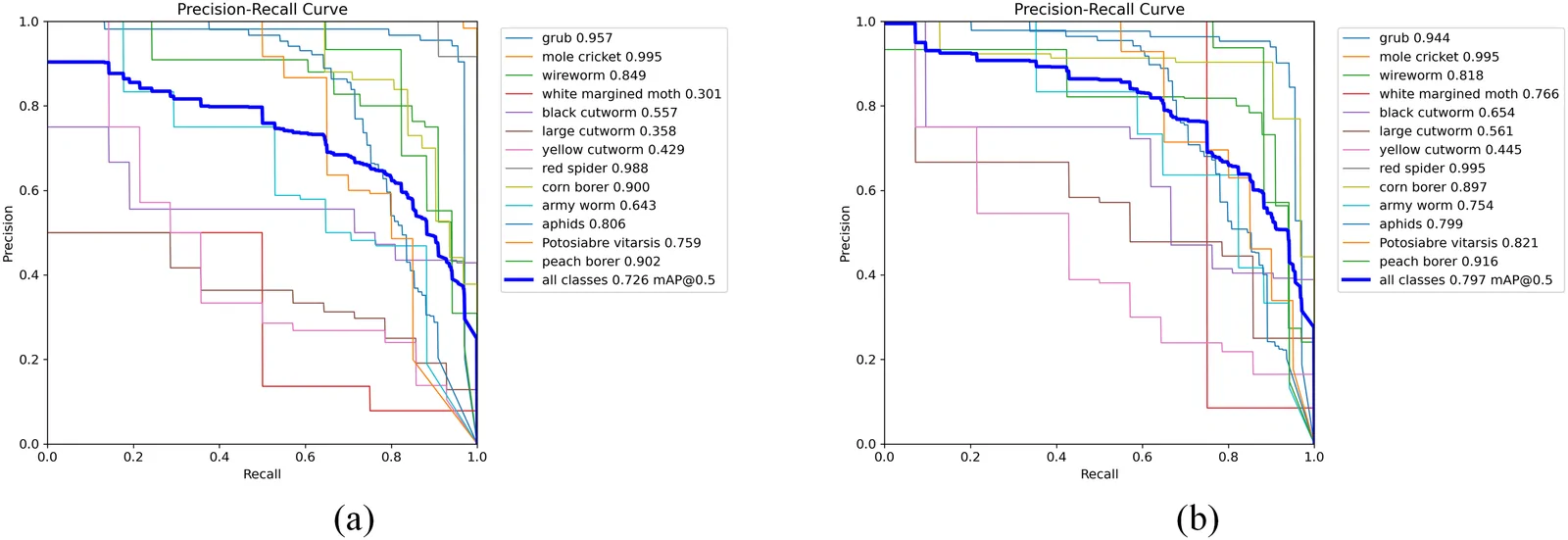
Precision-Recall (PR) curve. **(a)** Precision-Recall (PR) curve of YOLOv11n. **(b)** Precision-Recall (PR) curve of WSD-YOLO.

The Fig 9 presents the Precision–Recall (P–R) curves corresponding to YOLOv11n (Fig 9(a)) and WSD-YOLO (Fig 9(b)) on the IPMaize dataset, together with the AP@0.5 for each class and the overall mAP@0.5. Judging from the overall geometric distribution, almost all 13 P–R curves of WSD-YOLO are located above or in the upper-left region compared with the baseline curves. The mAP@0.5 improved from 72.6 % to 79.7 %, representing a relative gain of 7.1 %, which constitutes a significant improvement for agricultural pest-detection tasks.

The performance gains for small-sample and hard-sample categories are particularly pronounced: the AP for white-margined moth (*n* = 49) jumped from 0.301 to 0.766, an increase of 46.5 percentage points, with its P–R curve transitioning from a sharp drop to a gentle decline; the AP for large cutworm (*n* = 153) increased from 0.358 to 0.561, a gain of 20.3 percentage points. Absolute improvements in AP were also achieved for black cutworm, yellow cutworm, army worm, Potosia brevitarsis, and peach borer.

For categories with sufficient samples and a baseline AP > 0.80, WSD-YOLO further raised the upper limit of recall while maintaining high precision: the AP for both mole cricket and red spider reached 0.995, with their tail curves tending to level off, indicating that the SHLT module effectively suppresses backgrounds with repetitive textures.

The AP decreases observed for grub, wireworm, corn borer, and aphids are attributed to the high visual homogeneity between the pests’ body color/texture and the background (e.g., highlighted soil surfaces, leaf veins, leaf sheaths, tassels), resulting in weak edge discriminability. This leads to misclassification of background highlights, wrinkles, or intersecting leaf veins as pest bodies, consequently causing a reduction in AP values.

**Fig 10:**
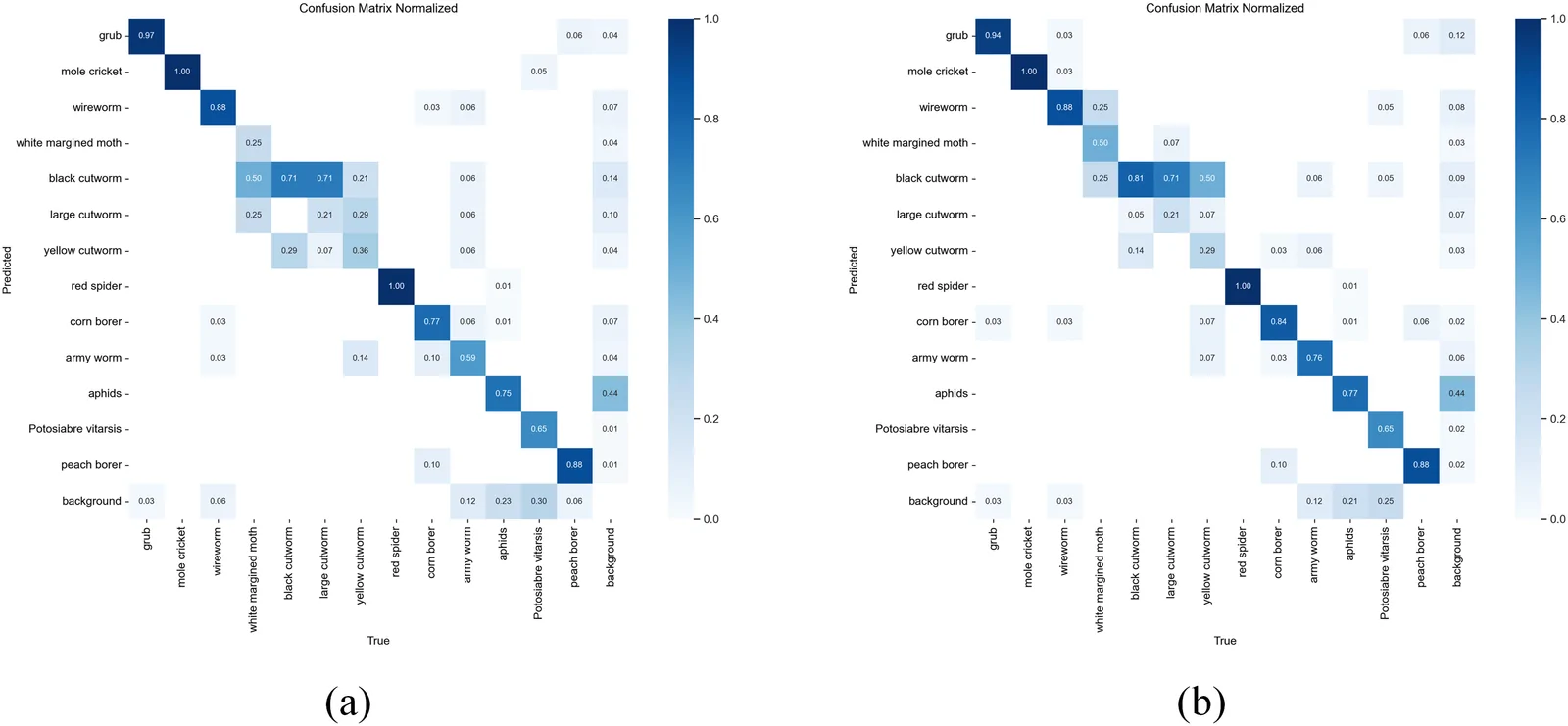
(a) Confusion matrix of YOLOv11n. **(b)** Confusion matrix of WSD-YOLO.

To further examine how WSD-YOLO differs from the baseline YOLOv11n in terms of performance, a comparison based on the confusion matrices of the two models was carried out, as shown in Fig 10. The confusion matrix, which visualizes the alignment between predicted categories and ground-truth categories, serves as an important tool for evaluating object detection models by intuitively reflecting the recognition accuracy and misclassification rates for each class.

The normalized confusion matrix for YOLOv11n exhibits distinct characteristics, notably showing high values for pests being missed and classified as background. This indicates a relatively pronounced issue with missed detections during the actual detection process. In contrast, the confusion matrix of WSD-YOLO shows a decreased proportion of pests being missed and classified as background. Overall, the model demonstrates more accurate predictions for the majority of categories and a lower missed detection rate. Notably, the recall for the white-margined moth improved from 25 % to 50 %, representing one of the most groundbreaking improvements in the enhanced model.

### 4.4 Comparison with other classic models

To assess the effectiveness and robustness of the proposed approach, our method was evaluated against a range of representative advanced and traditional detection models, including the YOLOv5 [45], YOLOv8 [46], YOLOv10 [20], YOLOv11 [21], YOLOv12 [31],RT-DETR-l [47], Hyper-YOLO [48], Maize-YOLO model [49], YOLOv9-t [50], RTMDet-tiny [51], PP-YOLOE-s [52], D-FINE-n [53]. As shown in Table 5, the WSD-YOLO model contains 3.8 M parameters and requires 9.2 GFLOPs, achieving an mAP@0.5 of 79.7%. Its mAP surpasses all other classic detection models, while its computational cost is only 1.5 times higher than that of the most efficient model in the comparison. Simultaneously, our model achieves an appropriate balance among speed, accuracy, and computational cost.

**Table 5:** Comparative experiments. Bold values indicate optimal results.

Model	Params(M)	Layer	P(%)	R(%)	mAP@0.5 (%)	GFLOPs	mAP@0.5:0.95 (%)	F1(%)	FPS	Inference time (ms/image)	Time(h)
YOLOv5n	2.5	193	70.4	70.2	74.1	7.1	49.4	70	106	9.4	1.382
YOLOv5s	9.1	193	69.8	75.2	76.7	23.9	51.3	72	99	10.1	1.762
YOLOv5m	25.1	248	67.7	74.1	75.1	64.0	49.9	70	78	12.8	2.526
YOLOv5l	53.1	303	66.1	74.9	72.3	134.7	46.8	70	52	19.2	4.867
YOLOv8n	3.0	**168**	61.9	73.4	73.9	8.1	48.3	66	130	7.7	**1.007**
YOLOv8s	11.1	168	66.2	75.7	75.9	25.8	50.6	71	106	9.4	1.390
YOLOv8m	25.8	218	71.0	71.1	76.0	78.7	51.5	71	68	14.7	2.513
YOLOv8l	43.6	268	68.2	74.3	75.3	164.9	50.9	70	49	20.4	4.715
YOLOv10n	2.7	285	72.1	65.9	73.0	8.3	49.3	68	**155**	**6.5**	1.561
YOLOv10s	8.0	285	69.0	64.8	71.5	24.5	48.6	66	119	8.4	2.390
YOLOv10m	16.5	369	70.2	66.5	72.1	63.5	50.0	68	87	11.5	4.331
YOLOv10l	25.7	467	**75.8**	61.6	71.5	126.1	48.7	66	59	17.0	15.823
YOLOv11n	2.6	238	69.5	71.6	72.6	6.3	48.2	70	118	8.5	1.387
YOLOv11s	9.4	238	70.5	71.1	75.0	21.3	49.9	71	103	9.7	1.520
YOLOv11m	20.0	303	70.1	72.4	74.7	67.7	50.4	71	73	13.7	3.197
YOLOv11l	25.3	464	65.9	76.1	73.9	86.6	48.8	70	64	15.6	3.658
YOLOv12n	2.5	376	66.0	69.2	73.0	**5.8**	47.9	66	123	8.1	1.649
YOLOv12s	9.1	376	69.1	72.8	74.6	19.3	49.9	69	91	11.0	4.105
YOLOv12m	19.6	402	68.2	69.1	72.8	59.5	47.5	68	55	18.2	9.475
RT-DETR-l	32.0	502	74.8	68.5	68.3	103.5	44.5	71	51	19.6	6.449
Hyper-YOLO-tiny	2.7	232	67.5	71.6	71.9	7.7	46.5	69	122	8.2	1.822
Hyper-YOLO-n	3.6	286	69.3	70.9	72.3	9.5	47.2	69	106	9.4	2.004
Hyper-YOLO-s	13.5	286	72.3	68.8	75.5	33.8	49.1	70	83	12.1	2.177
Hyper-YOLO-m	30.7	341	68.7	71.0	74.3	91.9	49.5	69	54	18.5	3.574
Maize-YOLO	33.4	527	73.3	77.3	76.3	38.9	51.2	**75**	67	14.9	10.598
YOLOv9-t	**2.0**	486	65.3	75.8	74.8	7.6	51.5	70	128	7.8	1.473
RTMDet-tiny	4.8	412	73.1	74.9	75.5	8.1	50.8	74	107	9.4	2.064
PP-YOLOE-s	7.9	364	72.5	74.2	74.8	17.4	50.3	73	93	10.8	3.134
D-FINE-n	3.9	326	73.1	74.4	75.3	7.1	50.9	74	114	8.8	2.960
WSD-YOLO	3.8	347	71.7	**77.4**	**79.7**	9.2	**52.7**	74	112	8.9	2.455

The results were obtained with seed = 42.

To further evaluate deployment efficiency, Table 6 compares the model size, peak GPU memory consumption, and inference latency of YOLOv11n and WSD-YOLO. All measurements were obtained using the best-performing weights with an input resolution of 640×640 and a batch size of 1 on a single NVIDIA GeForce RTX 3090 GPU.

**Table 6:** Deployment efficiency comparison between YOLOv11n and WSD-YOLO.

Structure	Params(M)	Model size(MB)	GFLOPs	Peak GPU Memory (MB)	Inference time(ms/image)	FPS
YOLOv11n (Baseline)	2.6	5.3	6.3	197.58	8.5	118
WSD-YOLO (Ours)	3.8	7.6	9.2	211.23	8.9	112

WSD-YOLO has a model size of 7.6 MB and requires 211.23 MB of peak GPU memory. Compared with YOLOv11n, the peak memory consumption increases by only 6.91%. Meanwhile, WSD-YOLO achieves an inference latency of 8.9 ms/image and a processing speed of 112 FPS, representing only a 4.71% latency increase over YOLOv11n. These results indicate that WSD-YOLO maintains a compact memory footprint and real-time inference capability, demonstrating its potential for agricultural pest monitoring on resource-constrained platforms.

### 4.5 Ablation and architectural analysis

To validate the impact of the proposed modules on maize pest detection performance and analyze the contribution of each component, ablation experiments were conducted on the IPMaize dataset. YOLOv11n was adopted as the baseline model, and the WMConv, SHLT, and DAWA modules were progressively incorporated into the network. Each configuration was independently trained three times using random seeds 42, 2026, and 3407 under identical experimental settings. Table 7 reports the mean ± sample standard deviation of the three runs, together with the corresponding model complexity (Params and GFLOPs).

**Table 7:** Ablation study results reported as mean ± standard deviation over three independent runs with random seeds 42, 2026, and 3407. Bold values indicate the optimal performance.

WMConv	SHLT	DAWA	P(%)	R(%)	mAP@0.5(%)	mAP@0.5:0.95(%)	F1(%)	Params(M)	GFLOPs
×	×	×	70.83 ± 1.17	69.80 ± 1.56	71.80 ± 0.75	48.17 ± 0.35	69.67 ± 0.58	2.6	6.3
✓	×	×	68.47 ± 1.69	73.10 ± 3.08	74.63 ± 1.00	50.00 ± 0.70	70.33 ± 1.15	**2.5**	**6.3**
×	✓	×	69.60 ± 2.17	72.97 ± 1.36	74.60 ± 0.10	49.63 ± 0.59	70.67 ± 1.15	3.2	6.6
×	×	✓	68.60 ± 2.96	74.53 ± 2.27	74.07 ± 1.15	48.87 ± 1.06	71.33 ± 1.53	3.1	6.6
✓	✓	×	69.63 ± 2.40	74.87 ± 2.89	76.07 ± 0.81	50.50 ± 0.62	71.67 ± 1.15	3.1	6.6
✓	×	✓	69.37 ± 5.42	73.13 ± 4.87	75.07 ± 0.40	50.17 ± 0.65	70.33 ± 0.58	3.1	8.9
×	✓	✓	71.43 ± 1.37	72.67 ± 0.83	76.23 ± 1.20	50.40 ± 0.61	72.67 ± 0.58	3.9	9.2
✓	✓	✓	**71.90 ± 0.26**	**76.90 ± 1.51**	**78.77 ± 0.83**	**52.63 ± 0.31**	**73.33 ± 0.58**	3.8	9.2

#### 4.5.1 Analysis of module effectiveness.

1. Effectiveness of WMConv

When only WMConv was introduced, the mean mAP@0.5 increased from 71.80% to 74.63%, while the mean recall improved from 69.80% to 73.10% over three independent runs. This indicates that WMConv, through its asymmetric convolutional structure and expanded receptive field, effectively enhances the model’s perception of multi-directional pest textures, particularly demonstrating significant improvements in recalling small-scale pests within complex backgrounds.

2. Effectiveness of SHLT

After incorporating SHLT alone, the mean mAP@0.5 increased to 74.60%, while the mean recall reached 72.97%. By introducing a self-attention mechanism on the low-resolution feature layer with the richest semantics, SHLT achieves efficient modeling of global context, effectively suppressing background interference such as repetitive leaf textures, thereby enhancing the model’s discriminative capability.

3. Effectiveness of DAWA

When only DAWA was introduced, the mean mAP@0.5 increased to 74.07%, and the mean recall reached 74.53%. Through its channel-spatial dual-path attention mechanism, DAWA integrates low-level detail information with high-level semantic representations, thereby enhancing the detection head’s capability to capture pest edges and texture characteristics, particularly excelling in scenarios where targets and backgrounds are similar.

#### 4.5.2 Module combination effects.

1. WMConv + SHLT

When WMConv and SHLT were combined, the mean mAP@0.5 further increased to 76.07%, while mAP@0.5:0.95 reached 50.50%. Meanwhile, the recall increased to 74.87%. This demonstrates the complementary nature of WMConv and SHLT in feature extraction and global modeling, with their combined action significantly enhancing the model’s overall detection capability.

2. WMConv + DAWA

This combination achieved a mean mAP@0.5 of 75.07%. Although the performance improvement was smaller than that of the WMConv+SHLT combination, the results suggest that WMConv and DAWA remain complementary in enhancing local texture representation and adaptive feature fusion.

3. SHLT + DAWA

The combination of SHLT and DAWA achieved a mean mAP@0.5 of 76.23% and an F1-score of 72.67%, further demonstrating the effectiveness of combining global semantic modeling with adaptive feature aggregation.

4. WMConv + SHLT + DAWA (WSD-YOLO)

When all three modules were jointly incorporated, WSD-YOLO achieved the best overall performance, with a mean mAP@0.5 of 78.77%, a mean mAP@0.5:0.95 of 52.63%, a mean Precision of 71.90%, a mean Recall of 76.90%, and a mean F1-score of 73.33%. Compared with the baseline YOLOv11n, the proposed model consistently achieved the highest detection accuracy while maintaining acceptable computational complexity. This fully proves the synergistic advantages of the proposed modules in feature extraction, contextual modeling, and multi-scale fusion, significantly improving the accuracy and robustness of maize pest detection.

#### 4.5.3 Architectural configuration sensitivity analysis.

To further verify the rationality of the final network configuration, additional sensitivity experiments were conducted from two perspectives: the insertion positions of WMConv and the integration strategy of the Transformer. For each comparison, only the investigated architectural component was changed, while the remaining network structure and training settings were kept unchanged.

1. Sensitivity to WMConv Insertion Positions

WMConv was inserted only in the Backbone, only in the Neck, and simultaneously in both the Backbone and Neck. The results are summarized in Table 8.

**Table 8:** Sensitivity analysis of WMConv insertion positions. Bold values indicate the optimal performance.

Configuration	P(%)	R(%)	mAP@0.5(%)	mAP@0.5:0.95(%)	F1(%)	Params(M)	GFLOPs
YOLOv11n(baseline)	**69.5**	71.6	72.6	48.2	70	2.6	6.3
WMConv in Backbone	65.1	76.1	75.4	49.5	70	2.5	6.3
WMConv in Neck	67.6	64.9	72.5	48.3	65	2.5	6.3
WMConv in Backbone and Neck (Ours)	66.6	**76.4**	**75.6**	**49.7**	**71**	**2.5**	**6.3**

Compared with YOLOv11n, inserting WMConv only in the Backbone increased mAP@0.5 from 72.6% to 75.4% and recall from 71.6% to 76.1%, indicating that WMConv is particularly effective during backbone feature encoding. In contrast, inserting WMConv only in the Neck produced an mAP@0.5 of 72.5% and reduced recall to 64.9%, suggesting that applying WMConv solely during feature fusion cannot fully exploit its directional representation capability. When WMConv was deployed in both the Backbone and Neck, the model achieved the highest mAP@0.5 of 75.6% and an F1-score of 71%, while maintaining 2.5 M parameters and 6.3 GFLOPs. These results support the placement adopted in the proposed architecture.

2. Comparison of Different Transformer Designs

To evaluate the single-scale design of SHLT, two alternative structures, namely SPPF + Transformer and a dual-scale Transformer, were implemented and compared with the original SPPF and SHLT. The results are presented in Table 9.

**Table 9:** Comparison of different transformer integration strategies. Bold values indicate the optimal performance.

Structure	P(%)	R(%)	mAP@0.5(%)	mAP@0.5:0.95(%)	F1(%)	Params(M)	GFLOPs
SPPF	69.5	71.6	72.6	48.2	70	**2.6**	**6.3**
SPPF + Transformer	68.0	69.2	73.7	48.5	68	3.1	6.5
Dual-scale-Transformer	70.4	67.9	73.9	48.4	69	5.4	7.4
SHLT (Ours)	**70.9**	**73.7**	**74.6**	**49.2**	**72**	3.2	6.6

Adding a Transformer after SPPF improved mAP@0.5 from 72.6% to 73.7%, confirming the benefit of global contextual modeling. The dual-scale Transformer further increased mAP@0.5 to 73.9%, but raised the parameter count to 5.4 M and the computational cost to 7.4 GFLOPs. In comparison, SHLT achieved the highest mAP@0.5 of 74.6%, precision of 70.9%, recall of 73.7%, and F1-score of 72%, with only 3.2 M parameters and 6.6 GFLOPs. These results demonstrate that applying self-attention only to the deepest semantic feature layer provides a better balance between detection accuracy and computational efficiency than the alternative Transformer designs.

Overall, the sensitivity experiments provide quantitative support for the module placement and Transformer configuration adopted in WSD-YOLO.

#### 4.5.4 Statistical validation and computational complexity analysis.

To further evaluate the reliability of the performance improvement and the computational cost introduced by the proposed modules, repeated experiments and complexity analysis were conducted. The baseline YOLOv11n and the proposed WSD-YOLO were independently trained three times using different random seeds (42, 2026, and 3407), while keeping all other training configurations unchanged. The experimental results are reported as mean ± standard deviation.

Since YOLOv11n and WSD-YOLO were evaluated under identical random seeds, a two-tailed paired Student’s *t*-test was employed to determine whether the observed improvements were statistically significant. A *p*-value smaller than 0.05 was considered statistically significant. The statistical significance test results are summarized in Table 10. The results demonstrate that WSD-YOLO consistently outperformed YOLOv11n across the major evaluation metrics. Specifically, the improvements in Recall (*p* = 0.0216), mAP@0.5 (*p* = 0.00016), mAP@0.5:0.95 (*p* = 0.0070), and F1-score (*p* = 0.0082) were statistically significant. In contrast, the improvement in Precision did not reach statistical significance (*p* = 0.2005). These results indicate that the proposed WSD-YOLO mainly enhances detection performance by improving feature representation and target retrieval capability.

**Table 10:** Statistical significance analysis between YOLOv11n and WSD-YOLO using paired Student’s t-test.

Metric	Mean difference (%)	p-value
Precision	+1.07	0.2005
Recall	+7.10	0.0216
mAP@0.5	+6.97	0.00016
mAP@0.5:0.95	+4.46	0.0070
F1	+3.67	0.0082

Furthermore, the repeated experiments show that WSD-YOLO maintains stable performance under different random initializations. The standard deviations of WSD-YOLO for Precision, Recall, mAP@0.5, mAP@0.5:0.95, and F1-score were 0.26%, 1.51%, 0.83%, 0.31%, and 0.58%, respectively. The relatively small variations among three independent runs demonstrate the reproducibility and robustness of the proposed method.

The computational complexity analysis further illustrates the trade-off between detection accuracy and model efficiency. Compared with YOLOv11n, the complete WSD-YOLO increased the number of parameters from 2.6 M to 3.8 M and GFLOPs from 6.3 to 9.2, corresponding to increases of approximately 46.2% and 46.0%, respectively. Meanwhile, the mean mAP@0.5 and mAP@0.5:0.95 were improved by 6.97 and 4.46 percentage points, respectively. These results indicate that the additional computational overhead introduced by WMConv, SHLT, and DAWA provides substantial accuracy improvements while maintaining the lightweight characteristics of the YOLOv11n framework.

Among the proposed modules, WMConv achieves the most favorable accuracy–complexity balance. It improves the mean mAP@0.5 by 2.83 percentage points while reducing the parameter number and maintaining the same computational complexity. SHLT introduces global contextual modeling with only a minor increase in computational cost, whereas DAWA provides stronger feature fusion capability at the expense of additional parameters and GFLOPs. Overall, WSD-YOLO achieves an effective balance between detection accuracy and computational efficiency, demonstrating its potential for practical maize pest detection applications.

#### 4.5.5 Comparison with representative modules.

To further verify the effectiveness of the proposed modules compared with representative feature enhancement methods, additional comparative experiments were conducted. Specifically, WMConv was compared with ACB and WTConv, SHLT was compared with LSKA and Mona, and DAWA was compared with CAFM and CGAFusion. All experiments were performed under identical training settings while replacing only the corresponding modules.

As shown in Table 11, the proposed WMConv, SHLT, and DAWA achieved better detection performance than their corresponding representative alternatives. Compared with ACB and WTConv, WMConv obtained higher accuracy, demonstrating that directional spatial modeling is more suitable for capturing irregular pest textures than conventional asymmetric convolution or frequency-domain feature extraction. SHLT achieved a better accuracy-efficiency trade-off than LSKA and Mona by introducing global contextual modeling only on the deepest semantic feature layer. Meanwhile, DAWA outperformed CAFM and CGAFusion, indicating that adaptive same-scale aggregation of complementary backbone and neck features is beneficial for maize pest detection.

**Table 11:** Comparison with representative modules.

Method	Params(M)	Layer	P(%)	R(%)	mAP@0.5(%)	GFLOPs	mAP@0.5:0.95(%)	F1(%)	FPS
WMConv	2.5	273	66.6	76.4	75.6	6.3	49.7	70	134
WTConv	3.2	301	63.7	77.6	73.6	7.5	48.9	69	111
ACB	2.5	258	66.3	76.2	75.0	6.2	49.6	70	118
SHLT	3.2	243	70.9	73.7	74.6	6.6	49.2	72	117
LSKA	2.9	244	67.7	71.1	73.1	6.5	49.5	68	130
Mona	2.5	242	67.0	71.0	73.4	6.2	49.7	67	112
DAWA	3.3	307	68.9	72.1	72.9	8.9	47.9	70	112
CGAFusion	3.0	280	66.8	68.1	71.7	8.7	48.1	67	113
CAFM	3.5	355	61.5	73.5	72.4	10.7	47.4	66	98

Overall, WMConv, SHLT, and DAWA improve feature representation from three complementary perspectives, namely local structural perception, global contextual modeling, and adaptive feature aggregation. Together with the conceptual comparison presented in Table 2, the controlled module-replacement experiments provide empirical support for the effectiveness of the proposed design choices relative to the selected representative methods. These findings further support the effectiveness and design rationale of the proposed WSD-YOLO architecture for maize pest detection.

### 4.6 Visualization of results

**Fig 11:**
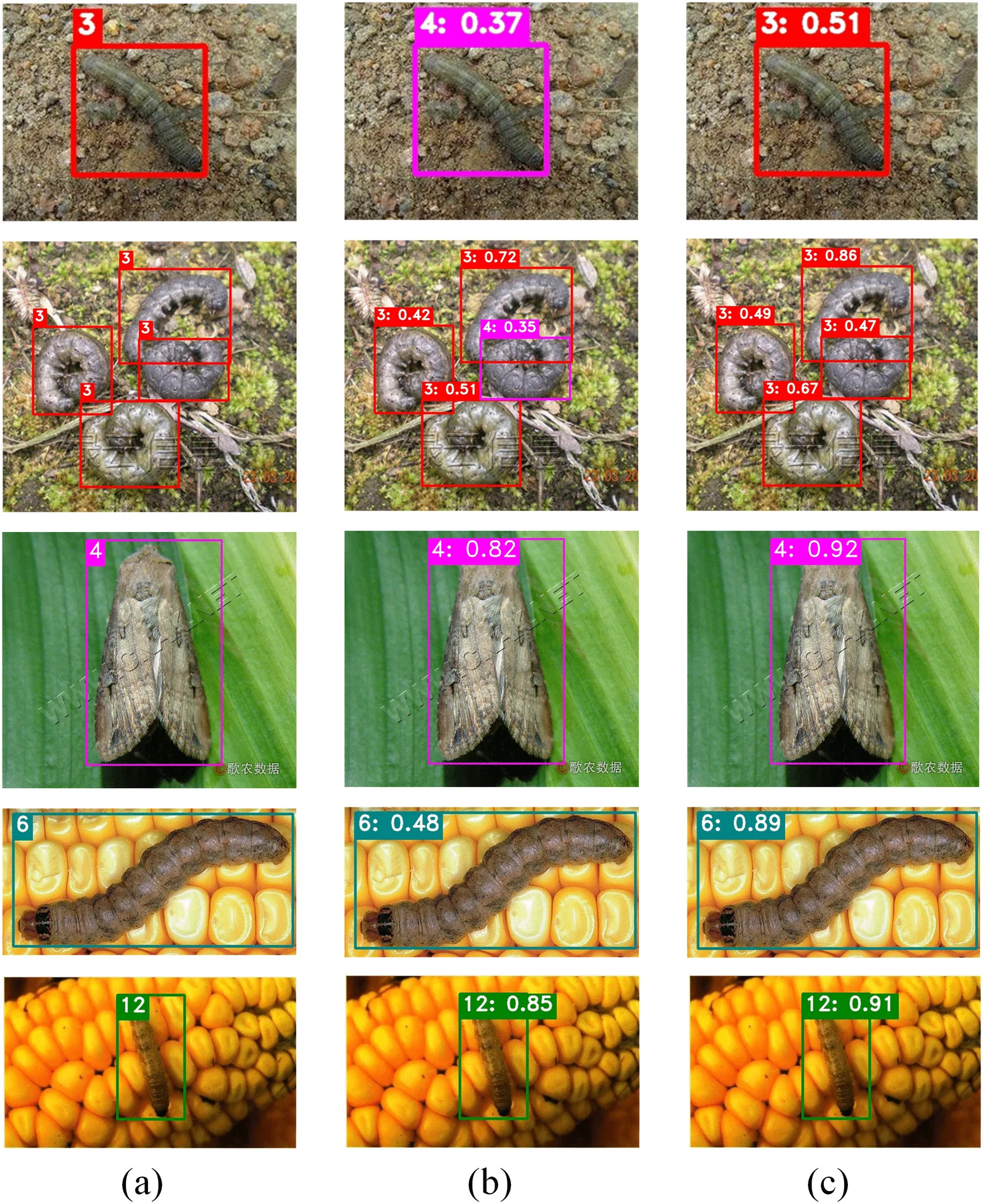
Visualization results on the IPMaize dataset. **(a)** Original images. **(b)** Detections by YOLOv11n. **(c)** Detections by WSD-YOLO.

To present a clearer comparison between the detection results of our proposed model and the baseline model YOLOv11n, we selected five representative sets of detection results from the IPMaize dataset for visualization, as shown in Fig 11.

As clearly shown in Fig 11, YOLOv11n still exhibits significant false detections for easily confusable categories. Comparing b(1), b(2) with c(1), c(2), it can be seen that the baseline YOLOv11n model misdetected category 3 (White margined moth) as category 4 (Black cutworm). However, WSD-YOLO not only corrected these misdetections but also achieved a relative increase in target confidence scores.

A comparison between b(3), b(4), b(5) and c(3), c(4), c(5) reveals that the confidence scores for targets detected by YOLOv11n were only 0.82, 0.48, and 0.85, respectively. In contrast, WSD-YOLO increased these confidence scores to 0.92, 0.89, and 0.91. This indicates that the WMConv convolution, the SHLT module, and the DAWA detail fusion module in WSD-YOLO effectively provide more discriminative textural features, enabling correct identification of the corresponding categories with significantly higher confidence. These improvements offer a more reliable decision-making basis for subsequent variable-rate pesticide application and pest infestation warnings in complex field environments.

### 4.7 Detection error analysis of AP-decreased categories

Although WSD-YOLO achieves overall performance improvements on the IPMaize dataset, slight AP degradation is observed for several categories, including Grub, Wireworm, Corn borer, and Aphids. To further investigate the underlying reasons for these performance variations, representative failure cases were visualized, and different error types were quantitatively counted.

**Fig 12:**
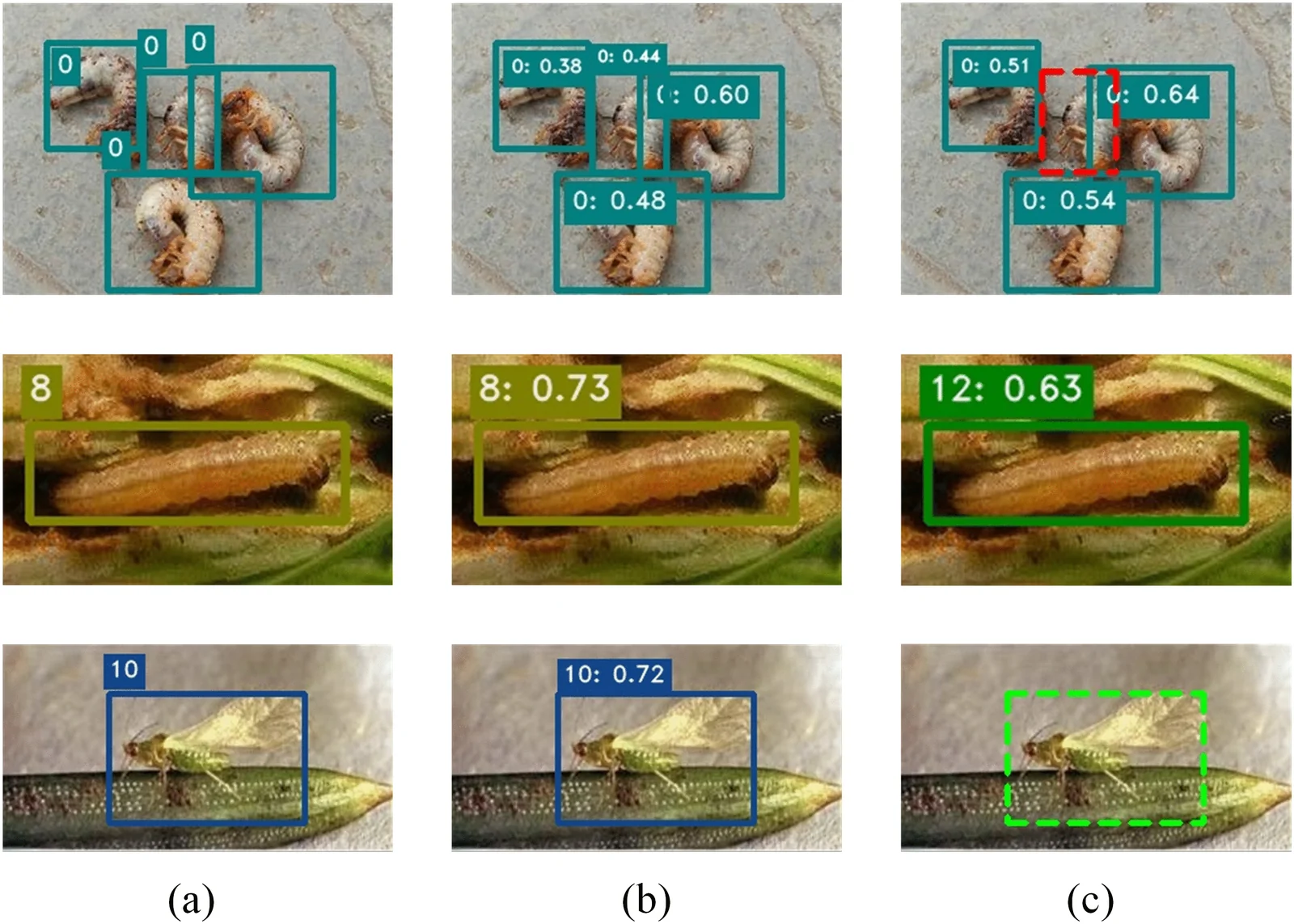
Visualization results on the IPMaize dataset. **(a)** Original images. **(b)** Detections by YOLOv11n. **(c)** Detections by WSD-YOLO. Red dashed boxes indicate background false positives, and green dashed boxes indicate false negatives.

Fig 12 presents representative detection error cases of YOLOv11n and WSD-YOLO for the AP-decreased categories. The first column shows the original images, while the second and third columns present the detection results of YOLOv11n and WSD-YOLO, respectively. Red dashed boxes indicate background false positives (BFP), where non-target background regions are incorrectly detected as pest targets. Green dashed boxes indicate false negatives (FN), where existing pest instances are missed by the detector.

To further quantify the sources of detection errors, three major error types were considered, including false negatives, background false positives, and category confusion. The statistical results are summarized in Table 12.

**Table 12:** Statistical analysis of different error types for AP-decreased pest categories.

Pest name	False Negative	Background False Positive	Category confusion
Grub	3	7	1
Wireworm	2	6	4
Corn borer	2	6	5
Aphids	22	38	2

As shown in Table 12, background false positives represent the most frequent error type among the AP-decreased categories. For Grub, Wireworm, Corn borer, and Aphids, 7, 6, 6, and 38 background false positive cases were observed, respectively. These errors mainly result from the visual similarity between pest targets and complex field backgrounds. Specifically, background structures such as soil textures, leaf veins, and crop residues may exhibit similar color or texture patterns to pest bodies, causing incorrect detections.

In addition, category confusion remains an important factor for some pest categories. Wireworm and Corn borer show relatively higher category confusion cases, with 4 and 5 errors, respectively. This indicates that morphological similarities among pest categories may introduce feature ambiguity during classification. Although WSD-YOLO improves feature representation through enhanced feature extraction and fusion mechanisms, distinguishing visually similar pest categories remains challenging under natural field conditions.

Aphids exhibit the highest detection difficulty, with 22 false negatives and 38 background false positives. This is mainly attributed to their extremely small size, weak boundaries, and frequent occurrence on leaf surfaces with complex textures. These characteristics make it difficult for the detector to extract sufficiently discriminative features and accurately localize pest instances.

Overall, the error analysis demonstrates that the AP degradation of several categories is mainly caused by intrinsic challenges in field-based pest detection, including weak target-background discrimination, small object characteristics, and inter-class morphological similarity. These findings further explain the remaining limitations of WSD-YOLO and provide guidance for future improvements in more challenging agricultural scenarios.

### 4.8 Validation of model generality

To assess the robustness and generalization of the proposed model, experiments were carried out using the Tomato Pest&Diseases dataset [54] and the IP102 dataset.

#### 4.8.1 Tomato Pest&Diseases Dataset.

**Fig 13:**
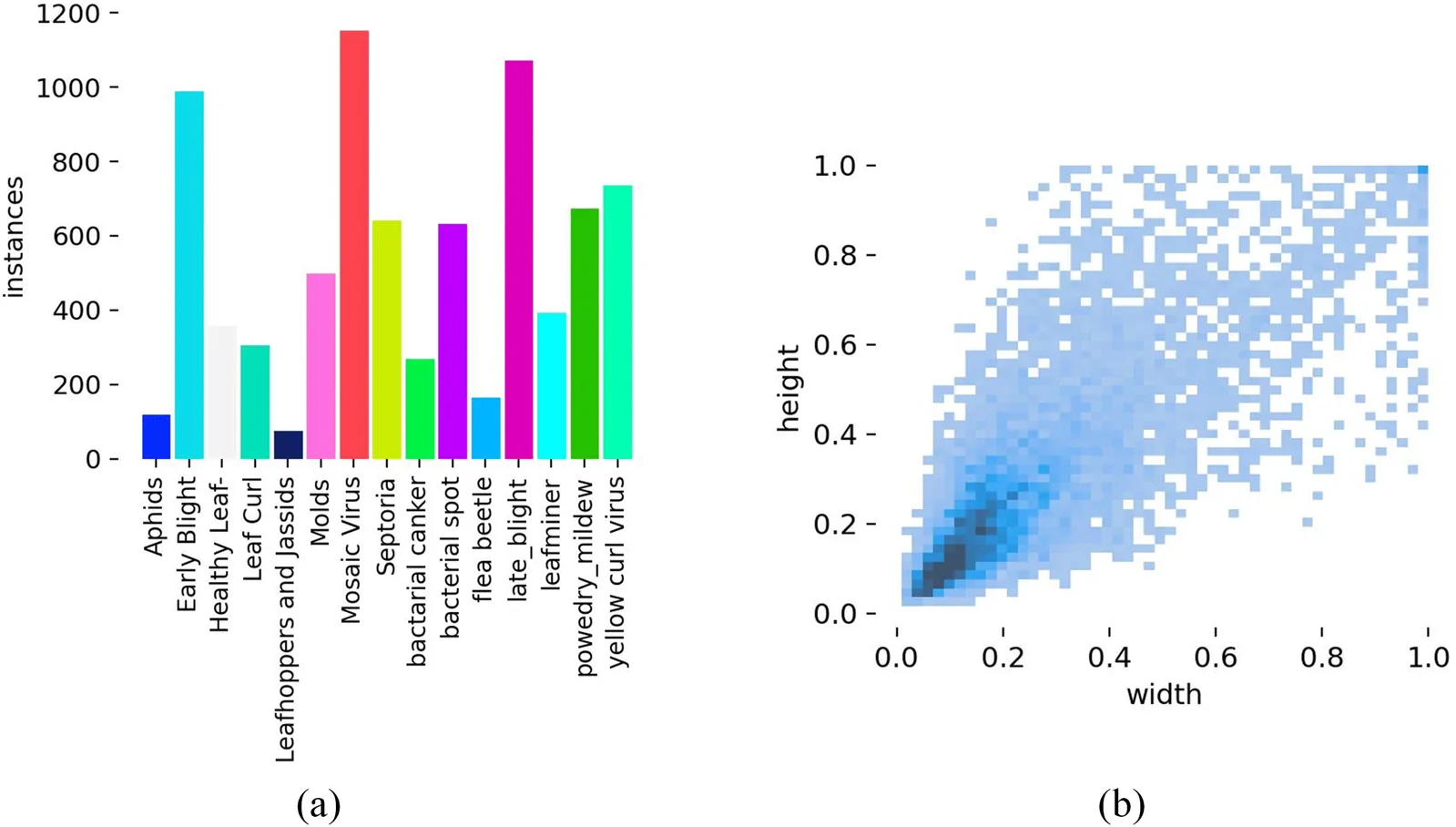
Distribution of 15 object categories.

The Tomato Pest&Disease Dataset contains a total of 2574 images representing 15 different types of tomato pests and diseases, as shown in the Fig 13. The high similarity among different pest and disease categories in the dataset poses a great challenge for accurate detection. The performance of WSD-YOLO and other models are summarized in Table 13. When compared to other existing models, the proposed WSD-YOLO model demonstrated superior detection performance, achieving improvements of 3.9% and 2.0% in mAP@0.5 and mAP@0.5:0.95, respectively, over the baseline model YOLOv11n.

**Table 13:** Comparative evaluation on the Tomato Pest&Diseases Dataset. Bold values indicate optimal results.

Model	mAP@0.5 (%)	mAP@0.5:0.95 (%)	P(%)	R(%)
YOLOv8n	29.3	13.3	37.7	29.8
YOLOv10n	28.9	14.2	36.1	29.1
YOLOv11n	30.5	14.4	37.4	30.8
YOLOv12n	31.3	12.6	30.3	**39.8**
Hyper-YOLO-tiny	31.2	14.2	37.0	36.2
YOLOv9-t	31.8	14.8	36.8	34.1
RTMDet-tiny	33.0	15.5	37.9	35.9
PP-YOLOE-s	32.5	15.2	37.3	35.4
WSD-YOLO	**34.4**	**16.4**	**38.4**	36.7

**Fig 14:**
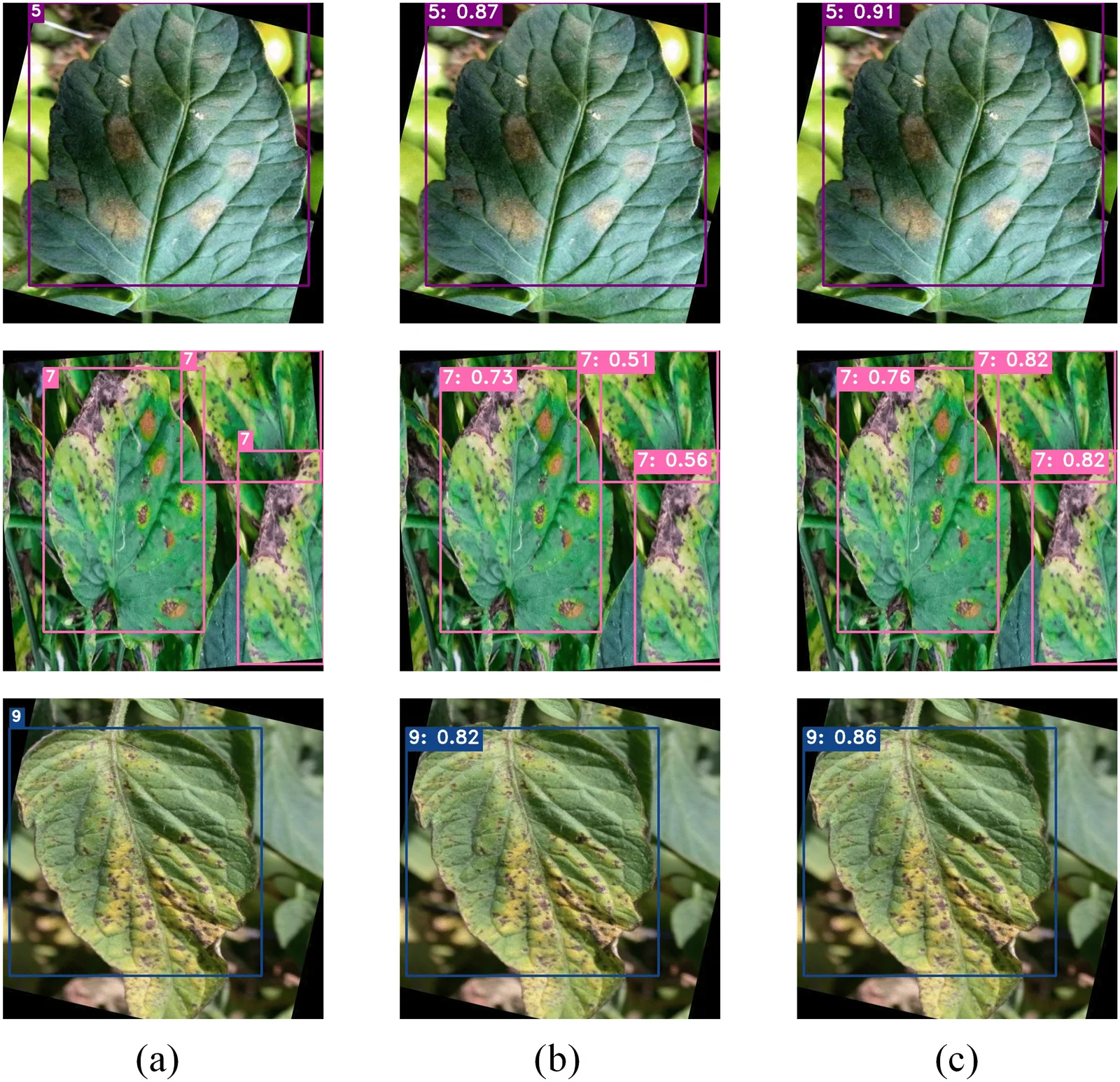
Visualization results on the Tomato Pest&Diseases dataset. **(a)** Original images. **(b)** Detections by YOLOv11n. **(c)** Detections by WSD-YOLO.

To provide a clear comparison between the performance of our model and the baseline YOLOv11n, three representative detection results from the Tomato Pest&Diseases dataset were selected, as shown in Fig 14. The results clearly demonstrate that WSD-YOLO outperforms YOLOv11n in this dataset. This comparison highlights the superior effectiveness and generalization ability of the proposed WSD-YOLO model on the Tomato Pest&Diseases dataset.

#### 4.8.2 IP102 dataset.

The IP102 dataset is a widely used pest image dataset, designed to provide a standardized platform for agricultural pest detection and recognition. It covers 102 unique pest categories, including a variety of common pests found in different crops and environments. The entire dataset consists of 18,975 images. The wide variety of pest categories and their high similarity in the dataset present substantial challenges for the accurate detection of the proposed model. When compared to other existing models, as shown in Table 14, the proposed WSD-YOLO model demonstrated superior detection performance, achieving improvements of 4.2% and 3.3% in mAP@0.5 and mAP@0.5:0.95, respectively, over the baseline model YOLOv11n.

**Table 14:** Comparative evaluation on the IP102 dataset. Bold values indicate optimal results.

Model	mAP@0.5 (%)	mAP@0.5:0.95 (%)	P(%)	R(%)
YOLOv8n	67.0	42.5	**58.6**	66.0
YOLOv10n	65.7	42.7	54.8	63.5
YOLOv11n	66.1	41.3	53.0	67.0
YOLOv12n	64.4	41.1	51.7	65.8
Hyper-YOLO-tiny	67.6	43.1	56.4	67.0
YOLOv9-t	68.6	43.7	56.5	68.1
RTMDet-tiny	68.0	43.3	55.9	67.4
PP-YOLOE-s	68.5	43.5	55.8	67.8
WSD-YOLO	**70.3**	**44.6**	56.6	**70.9**

**Fig 15:**
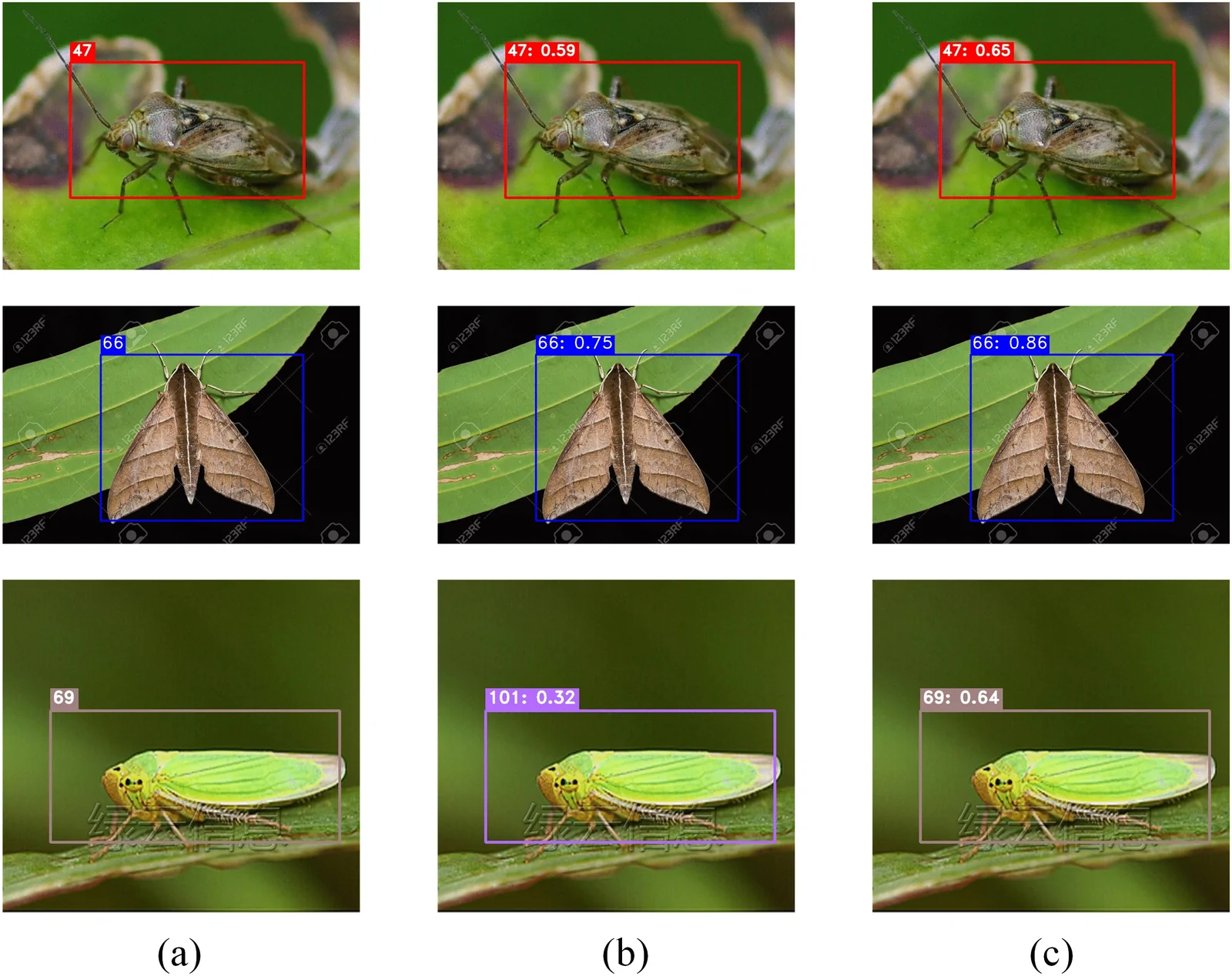
Visualization results on the IP102 dataset. **(a)** Original images. **(b)** Detections by YOLOv11n. **(c)** Detections by WSD-YOLO.

Three representative detection results on the IP102 dataset are illustrated in Fig 15. In the last group, YOLOv11n caused misdetection. Experimental results on the IP102 dataset demonstrate that our proposed WSD-YOLO model achieves favorable detection performance and strong generalization ability.

#### 4.8.3 Quantitative analysis of cross-dataset performance degradation.

Although WSD-YOLO was evaluated on the Tomato Pest&Diseases and IP102 datasets, its detection performance varies across datasets because of domain shift. Therefore, the relative performance degradation (PD) is introduced to quantitatively evaluate the performance difference between the source-domain IPMaize dataset and each target dataset. All results in this subsection are obtained using the same experimental configuration as the preceding cross-dataset experiments.


$$\begin{array}{c} PD(\%)=\frac{mAP_{\text{source}}-mAP_{\text{target}}}{mAP_{\text{source}}}\times 100\% \end{array}$$ (41)

where $mAP_{source}$ and $mAP_{target}$ denote the mAP@0.5 values obtained on IPMaize and the corresponding target dataset, respectively. A lower PD indicates better generalization under the evaluated target-domain conditions.

As shown in Table 15, the PD of WSD-YOLO is 56.84% on the Tomato Pest&Diseases dataset and 11.79% on the IP102 dataset. The larger degradation on Tomato Pest&Diseases can be attributed to its substantial differences from IPMaize in crop species, object categories, imaging conditions, background characteristics, and target appearance. In contrast, IP102 contains numerous agricultural pest categories with visual characteristics similar to those in IPMaize, resulting in a relatively smaller distribution discrepancy and lower performance degradation.

**Table 15:** Quantitative analysis of cross-dataset performance degradation between YOLOv11n and WSD-YOLO.

Model	Dataset	Source-domain mAP@0.5 (%)	Target-domain mAP@0.5 (%)	PD(%)
YOLOv11n	Tomato Pest&Diseases	72.6	30.5	57.99
WSD-YOLO	Tomato Pest&Diseases	79.7	34.4	56.84
YOLOv11n	IP102	72.6	66.1	8.95
WSD-YOLO	IP102	79.7	70.3	11.79

Despite domain-induced performance degradation, WSD-YOLO consistently achieves higher absolute detection accuracy than YOLOv11n on both target datasets. Its PD is slightly lower on the Tomato Pest&Diseases dataset, whereas a higher PD is observed on IP102 due to its substantially improved source-domain performance. These results suggest that WSD-YOLO maintains strong cross-dataset performance, although the relative performance retention varies across target domains.

## 5. Conclusion

This paper proposes WSD-YOLO, an improved maize pest detection model based on the YOLOv11n framework, designed to address the challenges of morphological variability and complex field backgrounds. By integrating three key modules—Windmill Convolution (WMConv) for multi-directional texture extraction, Single-Scale High-Level Transformer (SHLT) for global context modeling, and Dual-Attention Weighted Aggregation (DAWA) for adaptive multi-scale feature fusion—the proposed method improves the reliability of pest identification in real-world agricultural environments.

Experimental results from three repeated training runs (random seeds: 42, 2026, 3407) on the IPMaize dataset demonstrate that WSD-YOLO achieves an averaged mAP@0.5 of 78.77% (79.7% under seed = 42) and averaged mAP@0.5:0.95 of 52.63%, outperforming a series of mainstream and recently released lightweight detectors, while maintaining a compact architecture of 3.8 million parameters. Cross-dataset validation on the Tomato Pest&Diseases and IP102 datasets, with PD metric adopted to quantify domain performance degradation, further confirms its strong generalization capability, indicating its adaptability to diverse pest species and environmental conditions.

From the perspective of sustainable pest management, the improved detection performance supports more precise interventions, which may help reduce unnecessary pesticide use. In addition, its compact model size and inference efficiency suggest its potential for deployment on edge devices for real-time field monitoring, contributing to more environmentally friendly agricultural practices.

Nevertheless, the current feature enhancement process still exhibits some redundancy, which increases parameter usage. Besides, statistical error analysis reveals frequent false positives and false negatives for tiny pests with background-similar textures. Future work will focus on incorporating more lightweight attention mechanisms and model pruning strategies to improve efficiency while further enhancing detection performance.

## References

[1] Erenstein O, Jaleta M, Sonder K, Mottaleb KA, Prasanna BM. Global maize production, consumption and trade: trends and R&D implications. Food Secur. 2022;14(5):1295–319. doi: 10.1007/s12571-022-01288-7

[2] Luo N, Meng Q, Feng P, Qu Z, Yu Y, Liu DL, et al. China can be self-sufficient in maize production by 2030 with optimal crop management. Nat Commun. 2023;14(1):2637. doi: 10.1038/s41467-023-38355-2 37149677 PMC10164166

[3] Nyamutukwa S, Mvumi BM, Chinwada P. Sustainable management of fall armyworm, *Spodoptera frugiperda* (J.E. Smith): challenges and proposed solutions from an African perspective. Int J Pest Manag. 2024;70(2). doi: 10.1080/09670874.2022.2027549

[4] Deutsch CA, Tewksbury JJ, Tigchelaar M, Battisti DS, Merrill SC, Huey RB, et al. Increase in crop losses to insect pests in a warming climate. Science. 2018;361(6405):916–9. doi: 10.1126/science.aat3466 30166490

[5] Ding B, Tian Y, Guo X, Wang L, Tian X. Improving rice pest management through RP11: a scientifically annotated dataset for adult insect recognition. Life (Basel). 2025;15(6):910. doi: 10.3390/life15060910 40566562 PMC12194132

[6] Wei Q, Zhang X, Yang F. Future range shifts in major maize insect pests suggest their increasing impacts on global maize production. Insects. 2025;16(6):568. doi: 10.3390/insects16060568 40558999 PMC12193563

[7] Wang S, Xu D, Liang H, Bai Y, Li X, Zhou J, et al. Advances in deep learning applications for plant disease and pest detection: a review. Remote Sens. 2025;17(4):698. doi: 10.3390/rs17040698

[8] Tudi M, Ruan HD, Wang L, Lyu J, Sadler R, Connell D, et al. Agriculture development, pesticide application and its impact on the environment. Int J Environ Res Public Health. 2021;18(3):1112. doi: 10.3390/ijerph18031112 33513796 PMC7908628

[9] Kamilaris A, Prenafeta-Boldú FX. Deep learning in agriculture: a survey. Comput Electron Agric. 2018;147:70–90. doi: 10.1016/j.compag.2018.02.016

[10] Patil SP, Zambre RS. Classification of cotton leaf spot disease using support vector machine. Int J Eng Res. 2014;3(4):1511–4.

[11] Ayan E, Erbay H, Varçın F. Crop pest classification with a genetic algorithm-based weighted ensemble of deep convolutional neural networks. Comput Electron Agric. 2020;179:105809. doi: 10.1016/j.compag.2020.105809

[12] Arnal Barbedo JG. Plant disease identification from individual lesions and spots using deep learning. Biosyst Eng. 2019;180:96–107. doi: 10.1016/j.biosystemseng.2019.02.002

[13] Krizhevsky A, Sutskever I, Hinton GE. ImageNet classification with deep convolutional neural networks. In: Advances in neural information processing systems. vol. 25; 2012. p. 1097–105. Available from: https://proceedings.neurips.cc/paper/2012/hash/c399862d3b9d6b76c8436e924a68c45b-Abstract.html

[14] Brahimi M, Arsenovic M, Laraba S, Sladojevic S, Boukhalfa K, Moussaoui A. Deep learning for plant diseases: detection and saliency map visualisation. In: Human and machine learning: visible, explainable, trustworthy and transparent. Springer; 2018. p. 93–117. Available from: doi: 10.1007/978-3-319-90403-0_6

[15] Wang CY, Bochkovskiy A, Liao HYM. YOLOv7: trainable bag-of-freebies sets new state-of-the-art for real-time object detectors. Proceedings of the IEEE/CVF Conference on Computer Vision and Pattern Recognition; 2023. p. 7464–75. Available from: 10.1109/CVPR52729.2023.00721

[16] Redmon J, Divvala S, Girshick R, Farhadi A. You only look once: unified, real-time object detection. Proceedings of the IEEE Conference on Computer Vision and Pattern Recognition; 2016. p. 779–88. Available from: 10.1109/CVPR.2016.91

[17] Terven J, Córdova-Esparza D-M, Romero-González J-A. A comprehensive review of YOLO architectures in computer vision: from YOLOv1 to YOLOv8 and YOLO-NAS. MAKE. 2023;5(4):1680–716. doi: 10.3390/make5040083

[18] Yaseen M. What is YOLOv9: an in-depth exploration of the internal features of the next-generation object detector. arXiv:2409.07813; 2024. Available from: doi: 10.48550/arXiv.2409.07813

[19] Ge Z, Liu S, Wang F, Li Z, Sun J. YOLOX: exceeding YOLO series in 2021. arXiv:2107.08430; 2021. Available from: doi: 10.48550/arXiv.2107.08430

[20] Wang A, Chen H, Liu L, Chen K, Lin Z, Han J, et al. YOLOv10: real-time end-to-end object detection. arXiv:2405.14458; 2024. Available from: doi: 10.48550/arXiv.2405.14458

[21] Ultralytics. Ultralytics YOLO11. Software; 2024 [cited 2025 Jan 18]. Available from: https://github.com/ultralytics/ultralytics

[22] Roy AM, Bhaduri J. Real-time growth stage detection model for high degree of occultation using DenseNet-fused YOLOv4. Comput Electron Agric. 2022;193:106694. doi: 10.1016/j.compag.2022.106694

[23] Lawal MO. Tomato detection based on modified YOLOv3 framework. Sci Rep. 2021;11(1):1447. doi: 10.1038/s41598-021-81216-5 33446897 PMC7809275

[24] Zhang B, Zhang M, Chen Y. Crop pest identification based on spatial pyramid pooling and deep convolution neural network. Trans Chin Soc Agric Eng. 2019;35(19):209–15. doi: 10.11975/j.issn.1002-6819.2019.19.025

[25] Tian Y, Yang G, Wang Z, Li E, Liang Z. Detection of apple lesions in orchards based on deep learning methods of CycleGAN and YOLOV3-dense. J Sens. 2019;2019:1–13. doi: 10.1155/2019/7630926

[26] Wu X, Zhan C, Lai YK, Cheng MM, Yang J. IP102: a large-scale benchmark dataset for insect pest recognition. Proceedings of the IEEE/CVF Conference on Computer Vision and Pattern Recognition; 2019. p. 8779–88. Available from: 10.1109/CVPR.2019.00899

[27] Amarathunga DC, Grundy J, Parry H, Dorin A. Methods of insect image capture and classification: a systematic literature review. Smart Agric Technol. 2021;1:100023. doi: 10.1016/j.atech.2021.100023

[28] Peng H, Xu H, Gao Z, Zhou Z, Tian X, Deng Q, et al. Crop pest image classification based on improved densely connected convolutional network. Front Plant Sci. 2023;14:1133060. doi: 10.3389/fpls.2023.1133060 37077629 PMC10106646

[29] Wang X, Xiao Z, Deng Z. Swin Attention Augmented Residual Network: a fine-grained pest image recognition method. Front Plant Sci. 2025;16:1619551. doi: 10.3389/fpls.2025.1619551 40612592 PMC12222059

[30] Dong Z, Wei X, Wu Y, Guo J, Zeng Z. Enhanced pest recognition using multi-task deep learning with the discriminative attention multi-network. Appl Sci. 2024;14(13):5543. doi: 10.3390/app14135543

[31] Sapkota R, Meng Z, Churuvija M, Du X, Ma Z, Karkee M. Comprehensive performance evaluation of YOLOv12, YOLO11, YOLOv10, YOLOv9 and YOLOv8 on detecting and counting fruitlet in complex orchard environments. Agric Commun. 2026;4(1):100125. doi: 10.1016/j.agrcom.2026.100125

[32] Khanam R, Hussain M. YOLOv11: an overview of the key architectural enhancements. arXiv:2410.17725; 2024. Available from: doi: 10.48550/arXiv.2410.17725

[33] Wang K, Liu J, Cai X. C2PSA-enhanced YOLOv11 architecture: a novel approach for small target detection in cotton disease diagnosis. arXiv:2508.12219; 2025. Available from: doi: 10.48550/arXiv.2508.12219

[34] Lin TY, Dollár P, Girshick R, He K, Hariharan B, Belongie SJ. Feature pyramid networks for object detection. Proceedings of the IEEE Conference on Computer Vision and Pattern Recognition; 2017. p. 936–44. Available from: 10.1109/CVPR.2017.106

[35] Liu S, Qi L, Qin H, Shi J, Jia J. Path aggregation network for instance segmentation. Proceedings of the IEEE/CVF Conference on Computer Vision and Pattern Recognition; 2018. p. 8759–68. Available from: 10.1109/cvpr.2018.00913

[36] Khasawneh N, Fraiwan M, Fraiwan L. Detection of K-complexes in EEG signals using deep transfer learning and YOLOv3. Clust Comput. 2022;26(6):3985–95. doi: 10.1007/s10586-022-03802-0

[37] Zhao J, Yang Z, Li B, Zhao Y. YOLO-DCRCF: an algorithm for detecting the wearing of safety helmets and gloves in power grid operation environments. J Imaging. 2025;11(9):320. doi: 10.3390/jimaging11090320 41003370 PMC12470609

[38] Felzenszwalb PF, Girshick RB, McAllester D, Ramanan D. Object detection with discriminatively trained part-based models. IEEE Trans Pattern Anal Mach Intell. 2010;32(9):1627–45. doi: 10.1109/TPAMI.2009.167 20634557

[39] Ding X, Guo Y, Ding G, Han J. ACNet: strengthening the kernel skeletons for powerful CNN via asymmetric convolution blocks. Proceedings of the IEEE/CVF International Conference on Computer Vision; 2019. p. 1911–20. Available from: 10.1109/ICCV.2019.00200

[40] Finder SE, Amoyal R, Treister E, Freifeld O. Wavelet convolutions for large receptive fields. Computer Vision – ECCV 2024. vol. 15112. Springer; 2024. p. 363–80. Available from: 10.1007/978-3-031-72949-2_21

[41] Lau KW, Po LM, Ur Rehman YA. Large separable kernel attention: rethinking the large kernel attention design in CNN. Expert Syst Appl. 2023;236:121352. doi: 10.1016/j.eswa.2023.121352

[42] Yin D, Hu L, Li B, Zhang Y, Yang X. 5%>100%: breaking performance shackles of full fine-tuning on visual recognition tasks. Proceedings of the IEEE/CVF Conference on Computer Vision and Pattern Recognition (CVPR); 2025. p. 20071–81. Available from: https://openaccess.thecvf.com/content/CVPR2025/html/Yin_5100_Breaking_Performance_Shackles_of_Full_Fine-Tuning_on_Visual_Recognition_CVPR_2025_paper.html

[43] Hu S, Gao F, Zhou X, Dong J, Du Q. Hybrid convolutional and attention network for hyperspectral image denoising. IEEE Geosci Remote Sens Lett. 2024;21:1–5. doi: 10.1109/lgrs.2024.3370299

[44] Chen Z, He Z, Lu Z-M. DEA-Net: single image dehazing based on detail-enhanced convolution and content-guided attention. IEEE Trans Image Process. 2024;33:1002–15. doi: 10.1109/TIP.2024.3354108 38252568

[45] Zhao Y, Ju Z, Sun T, Dong F, Li J, Yang R, et al. TGC-YOLOv5: an enhanced YOLOv5 drone detection model based on transformer, GAM & CA attention mechanism. Drones. 2023;7(7):446. doi: 10.3390/drones7070446

[46] Ultralytics. Ultralytics YOLOv8. Software; 2023 [cited 2025 Jan 18]. Available from: https://github.com/ultralytics/ultralytics

[47] Zhao Y, Lv W, Xu S, Wei J, Wang G, Dang Q, et al. DETRs beat YOLOs on real-time object detection. Proceedings of the IEEE/CVF Conference on Computer Vision and Pattern Recognition; 2024. p. 16965–74. Available from: 10.1109/CVPR52733.2024.01605

[48] Feng Y, Huang J, Du S, Ying S, Yong J-H, Li Y, et al. Hyper-YOLO: when visual object detection meets hypergraph computation. IEEE Trans Pattern Anal Mach Intell. 2025;47(4):2388–401. doi: 10.1109/TPAMI.2024.3524377 40030788

[49] Yang S, Xing Z, Wang H, Dong X, Gao X, Liu Z, et al. Maize-YOLO: a new high-precision and real-time method for maize pest detection. Insects. 2023;14(3):278. doi: 10.3390/insects14030278 36975962 PMC10051432

[50] Wang CY, Yeh IH, Liao HYM. YOLOv9: learning what you want to learn using programmable gradient information. Computer Vision – ECCV 2024. vol. 15089. Springer; 2024. p. 1–21. Available from: 10.1007/978-3-031-72751-1_1

[51] Lyu C, Zhang W, Huang H, Zhou Y, Wang Y, Liu Y, et al. RTMDet: an empirical study of designing real-time object detectors. arXiv:2212.07784; 2022. Available from: doi: 10.48550/arXiv.2212.07784

[52] Xu S, Wang X, Lv W, Chang Q, Cui C, Deng K, et al. PP-YOLOE: an evolved version of YOLO. arXiv:2203.16250; 2022. Available from: doi: 10.48550/arXiv.2203.16250

[53] Peng Y, Li H, Wu P, Zhang Y, Sun X, Wu F. D-FINE: redefine regression task of DETRs as fine-grained distribution refinement. International Conference on Learning Representations; 2025. Available from: https://openreview.net/forum?id=MFZjrTFE7h

[54] Absolute. Tomato pest & diseases dataset. Roboflow Universe; 2023 [cited 2025 Jan 18]. Available from: https://universe.roboflow.com/absolute/tomato-pest-diseases

